# Genetic characterization of the cyclohexane carboxylate degradation pathway in the denitrifying bacterium *Aromatoleum* sp. CIB

**DOI:** 10.1111/1462-2920.16093

**Published:** 2022-06-29

**Authors:** David Sanz, Eduardo Díaz

**Affiliations:** ^1^ Department of Microbial and Plant Biotechnology Centro de Investigaciones Biológicas Margarita Salas‐CSIC Madrid Spain

## Abstract

The alicyclic compound cyclohexane carboxylate (CHC) is anaerobically degraded through a peripheral pathway that converges with the central benzoyl‐CoA degradation pathway of aromatic compounds in *Rhodopseudomonas palustris* (bad pathway) and some strictly anaerobic bacteria. Here we show that in denitrifying bacteria, e.g. *Aromatoleum* sp. CIB strain, CHC is degraded through a bad‐ali pathway similar to that reported in *R*. *palustris* but that does not share common intermediates with the benzoyl‐CoA degradation pathway (bzd pathway) of this bacterium. The *bad‐ali* genes are also involved in the aerobic degradation of CHC in strain CIB, and orthologous *bad‐ali* clusters have been identified in the genomes of a wide variety of bacteria. Expression of *bad‐ali* genes in strain CIB is under control of the BadR transcriptional repressor, which was shown to recognize CHC‐CoA, the first intermediate of the pathway, as effector, and whose operator region (CAAN_4_TTG) was conserved in *bad‐ali* clusters from Gram‐negative bacteria. The bad‐ali and bzd pathways generate pimelyl‐CoA and 3‐hydroxypimelyl‐CoA, respectively, that are metabolized through a common aab pathway whose genetic determinants form a supraoperonic clustering with the *bad*‐*ali* genes. A synthetic *bad‐ali‐aab* catabolic module was engineered and it was shown to confer CHC degradation abilities to different bacterial hosts.

## INTRODUCTION

**FIGURE 1:**
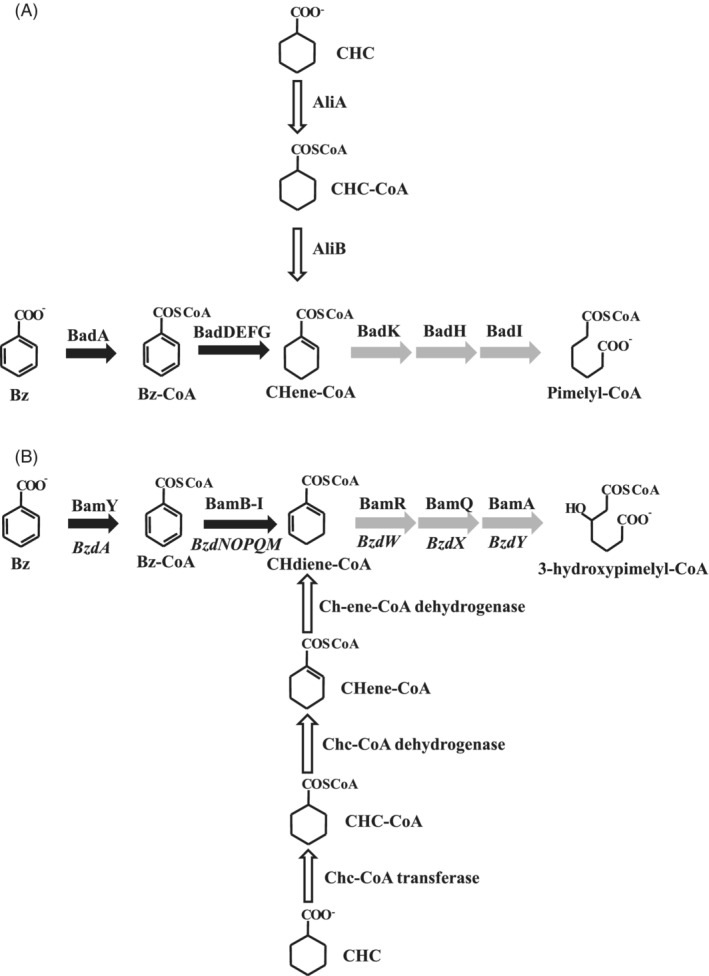
Scheme of known CHC degradation pathways in anaerobic bacteria. (A) CHC degradation in the photosynthetic *R*. *palustris*. (B) CHC degradation in *G*. *metallireducens* and other strict anaerobes. The enzymes involved in anaerobic benzoate degradation in *Aromatoleum* sp. CIB are also indicated (in italics). Abbreviations: Bz, benzoate; Bz‐CoA, benzoyl‐CoA; CHC, cyclohexane carboxylate; CHC‐CoA, cyclohexane carboxyl‐CoA; CHeneCoA, cyclo‐hex‐1‐ene‐1‐carboxyl‐CoA; CHdieneCoA, cyclohexa‐1,5‐diene‐1‐carboxyl‐CoA. Black arrows: enzymes involved in anaerobic benzoate degradation; white arrows, enzymes involved in CHC degradation; grey arrows, enzymes involved in anaerobic degradation of benzoate and CHC

The alicyclic compound cyclohexane carboxylate (CHC) is present in the environment where it is synthesized as a functional moiety of plant secondary products (Floss et al., 1992), as part of polyketide antibiotics produced by some microorganisms, e.g. *Streptomyces* species (Cropp et al., 2000), or as a metabolite produced during fermentation of benzoate or crotonate (Boll et al., 2016; Kung et al., 2013; Mouttaki et al., 2007). CHC plays an important role in the modern chemical industry because it is involved in drug or pesticides production. As a consequence of its industrial use, CHC is released into the environment in sewage of petrochemical production plants, or during the refining of crude oil as a main component of naphthenic acids (Wang et al., 2016; Whitby, 2010). Microbial degradation of CHC represents an eco‐friendly strategy to remove this compound from the environment. Several strategies to degrade CHC have been described both in aerobic and anaerobic bacteria, and all of them reveal a remarkable connection with the catabolism of aromatic compounds. Under oxic conditions, the CHC degradation pathway, reported in *Alcaligenes*, *Arthrobacter*, *Corynebacterium (Sinomonas)*, *Acinetobacter*, *Alkanivorax* and *Pseudomonas* strains (Blakley, 1974; Blakley & Papish, 1982; Kaneda, 1974; Smith & Callely, 1975; Taylor & Trudgill, 1978; Whitby, 2010; Yamamoto et al., 2021), generates 4‐hydroxybenzoate. The aromatization of CHC is initiated through hydroxylation at the 4‐position by a P450 monooxygenase system generating 4‐hydroxy‐CHC, which following dehydrogenation to form 4‐oxo‐CHC is then aromatized to 4‐hydroxybenzoate by the action of two consecutive desaturases (Δ dehydrogenases) (Yamamoto et al., 2021). These bacteria were able to metabolize 4‐hydroxybenzoate yielding the central intermediate protocatechuate, which is further degraded via the β‐ketoadipate pathway. Under anoxic conditions, two different CHC catabolic pathways have been characterized in the facultatively anaerobic photosynthetic α‐proteobacterium *Rhodopseudomonas palustris* (Küver et al., 1995) and in the strict anaerobic δ‐proteobacterium *Geobacter metallireducens* (Kung et al., 2014). In *R*. *palustris* the CHC metabolism produces cyclo‐hex‐1‐ene‐1‐carboxyl‐CoA (CHene‐CoA) by the action of a specific CoA ligase (AliA) and the further oxidation catalysed by the acyl‐CoA dehydrogenase AliB [Figure 1(A)] (Egland et al., 1997; Samanta & Harwood, 2005). CHene‐CoA is also the product of the reaction catalysed by the class I benzoyl‐CoA reductase (BadDEFG) during the anaerobic degradation of benzoate in *R*. *palustris*, thus being the common intermediate of the degradation of CHC and aromatic compounds in this bacterium [Figure 1(A)]. The CHene‐CoA is further subjected to modified β‐oxidation through hydration, oxidation and hydrolytic cleavage by BadK, BadH and BadI enzymes, respectively, yielding the aliphatic pimelyl‐CoA [Figure 1(A)] (Egland et al., 1997; Pelletier & Harwood, 1998; Pelletier & Harwood, 2000; Perrotta & Harwood, 1994). Pimelyl‐CoA is then metabolized through a β‐oxidation of dicarboxylic acids to yield three acetyl‐CoA molecules and one CO_2_ (Harrison & Harwood, 2005). Thus, in *R*. *palustris* the bad‐ali pathway, encoded by the *bad‐ali* cluster, is shared for the anaerobic degradation of both CHC and aromatic compounds [Figure 1(A)]. Since homologous *ali* and *bad* genes have been described in some *Rhodococcus* and *Cupriavidus* strains, a modified β‐oxidation pathway was also proposed to be involved in aerobic CHC degradation in these microorganisms (Presentato et al., 2018; Wang et al., 2015; Zampolli et al., 2020). In *G*. *metallireducens* the anaerobic CHC metabolism involves first a succinyl‐CoA:CHC CoA transferase to activate CHC to CHC‐CoA, then a CHC‐CoA dehydrogenase catalyses the 1,2‐dehydrogenation of CHC‐CoA to CHene‐CoA, and finally a cyclohexa‐1,5‐diene‐1‐carboxyl‐CoA dehydrogenase catalyses a 1,4‐dehydrogenation to produce cyclohexa‐1,5‐diene‐1‐carboxyl‐CoA (CHdieneCoA) [Figure 1(B)] (Kung et al., 2014). CHdieneCoA is also generated during the anaerobic benzoate degradation catalysed by the ATP‐independent class II benzoyl‐CoA reductase (Kung et al., 2009), so this metabolite is the joint intermediate of the CHC and benzoate anaerobic catabolism in *G*. *metallireducens* [Figure 1(B)]. Further degradation of CHdieneCoA through a modified β‐oxidation pathway that yields 3‐hydroxypimelyl‐CoA is carried out by a set of enzymes, i.e. a hydratase (BamR), dehydrogenase (BamQ) and ring‐cleaving hydrolase (BamA), that differ from those of *R*. *palustris* but that are conserved in all other anaerobic benzoate degraders [Figure 1(B)] (Durante‐Rodríguez et al., 2018). The chc pathway that converts CHC into CHdieneCoA in *G*. *metallireducens* was postulated to be present in all CHC‐degrading anaerobic bacteria, e.g. denitrifying, Fe(III)‐reducing, sulfate‐reducing and fermenting bacteria, other than *R*. *palustris* (Kung et al., 2013; Kung et al., 2014). Nevertheless, despite *R*. *palustris* and strict anaerobes use two different central pathways for CHC catabolism, both central pathways finally converge in a common (3‐hydroxy)‐pimelyl‐CoA degradation route (lower pathway).

*Azoarcus* sp. CIB (currently reclassified as *Aromatoleum* sp. CIB (Rabus et al., 2019; Raittz et al., 2021) is a facultative anaerobic β‐proteobacterium able to degrade either aerobically or anaerobically (denitrifying) a wide range of aromatic compounds. Under anoxic conditions most aromatic compounds are funnelled to the benzoyl‐CoA central intermediate that is converted to the aliphatic 3‐hydroxypimelyl‐CoA compound via the Bzd pathway encoded by the *bzd* cluster (Carmona et al., 2009; Durante‐Rodríguez et al., 2018; López‐Barragán et al., 2004; Valderrama et al., 2019) [Figure 1(B)]. The strain CIB is also able to grow anaerobically using CHC as sole carbon and energy source (Blázquez et al., 2008). Since a *bad‐ali* cluster similar to the one described in *R*. *palustris* was predicted in the genome of *Aromatoleum* sp. CIB (Martín‐Moldes et al., 2015), this strain constitutes a good model system to study the genetic determinants of CHC degradation in denitrifying bacteria. Interestingly, this work reveals for the first time that the anaerobic degradation of aromatic compounds (benzoate) and CHC follows different central pathways in denitrifying bacteria. A lower pathway (*aab* cluster) for pimelyl‐CoA (and 3‐hydroxypimelyl‐CoA) conversion to glutaryl‐CoA has been also identified in strain CIB, and a recombinant cassette which encodes the complete CHC catabolic pathway (*bad‐aab* genes) has been engineered and successfully used to expand the catabolic abilities of several biotechnologically relevant bacteria towards the alicyclic CHC compound.

## EXPERIMENTAL PROCEDURES

### Bacterial strains, plasmids and growth conditions

The bacterial strains as well as the plasmids used in this work are listed in Table 1. *Escherichia coli* cells were usually grown at 37°C in lysogeny broth (LB) medium (Miller, 1972), or in MC minimal medium (López‐Barragán et al., 2004) using 0.2% glucose as carbon source. *Aromatoleum* and *Azoarcus* strains were grown anaerobically at 30°C in MC medium as described previously using the indicated carbon source(s) and 10 mM nitrate as the terminal electron acceptor (López‐Barragán et al., 2004). *Aromatoleum*, *Azoarcus*, *Acinetobacter*, *Paraburkholderia* and *Pseudomonas* strains were grown aerobically at 30°C in NB medium (Difco, 234000) or in MC minimal medium supplemented with the indicated carbon source but without nitrate. When needed, antibiotics were added to the culture medium at the following concentrations: gentamicin (7.5 μg/ml), kanamycin (50 μg/ml). Growth was determined by measuring absorbance at 600 nm (*A*_600_) in a Shimadzu UV‐260 spectrophotometer.

**TABLE 1:** Bacterial strains and plasmids used in this work

Strain or plasmid	Relevant genotype and main characteristics	Reference or source
***E*. *coli* strains**		
DH10B	*F'*, *mcrA*, *Δ(mrr hsdRMS‐mcrBC)*, *Φ80lacZΔM15*, *ΔlacX74*, *deoR*, *recA1*, *araD139*, *Δ(ara‐leu)7697*, *galU*, *galK*, *rpsL* (Sm^R^), *endA1*, *nupG*	Life Technologies
S17‐1λpir	Tp^r^ Sm^r^ *recA thi hsdRM* ^+^ *RP42:*:.*Tc::Mu::Km Tn7 λpir* phage lysogen	de Lorenzo and Timmis (1994)
BL21 (DE3)	F^−^, *ompT*, *hsdS*_*B*_(r_B_^−^ m_B_^−^), *gal*, *dcm*, λDE3	Sambrook and Russell (2001)
MC4100	*araD139* Δ(*argF‐lac*)U169 *rpsL* 150 (Sm^r^) *relA1 flbB*5301 *deo*C1 *ptsF25 rbsR*	Casadaban (1976)
JW3375‐1	F^−^, *Δ(araD‐araB)567*, *ΔlacZ4787*(::rrnB‐3), *λ* ^*−*^ *ΔbioH756*::*kan*, *rph‐1*, *Δ(rhaD‐rhaB)568*, *hsdR514*	Baba et al. (2006)
***Aromatoleum/Azoarcus* strains**		
*Aromatoleum* sp. CIB	Wild‐type strain (previously named as *Azoarcus* sp. CIB)	López‐Barragán et al. (2004)
*Aromatoleum* sp. CIBΔbadHI	CIB mutant strain with a deletion of the *badHI* genes	This work
*Aromatoleum* sp. CIBΔAzCIB_1938	CIB mutant strain with a deletion of the *aabC* gene	This work
*Azoarcus communis* SWub3	Wild‐type strain (LMG22127)	Reinhold‐Hurek et al. (1993)
***Paraburkholderia* strains**		
*Paraburkholderia xenovorans* LB400	Wild‐type strain	Denef et al. (2004)
***Acinetobacter* strains**		
*Acinetobacter baylyi* ADP1	Wild‐type strain	Juni and Janik (1969)
***Pseudomonas* strains**		
*P*. *putida* KT2440	Wild‐type strain	Franklin et al. (1981)
**Plasmids**		
pIZ1016	Gm^r^, pBBR1MCS‐5 Mob^+^, *lacZα*, *Ptac/lacI* ^*q*^ broad‐host range cloning vector	Moreno‐Ruiz et al. (2003)
pIZ2	Gm^r^, pIZ1016 derivative with an extended polylinker	Acedos et al. (2021)
pIZBad	Gm^r^, pIZ2 derivative expressing a synthetic *bad‐ali* cassette (aliB*aliA*badK*badH*badI*) from *Aromatoleum* sp. CIB under control of *lacI* ^q^/*Ptac*	This work
pIZBad_A	Gm^r^, pIZ2 derivative expressing an extended *bad‐ali* cassette containing the *aabA* gene from *Aromatoleum* sp. CIB under control of *lacI* ^q^/*Ptac*	This work
pIZBadβ1	Gm^r^, pIZ2 derivative expressing the synthetic cluster *bad‐ali* with *aabA* and the *aabBCD* genes from *Aromatoleum* sp. CIB under control of *lacI* ^q^/*Ptac*	This work
pIZBadR	Gm^r^, pIZ2 derivative expressing *badR* gene from strain CIB under control of *lacI* ^q^/*Ptac*	This work
pIZBadRAliA	Gm^r^, pIZ2 derivative expressing the *badR* and *aliA* genes from strain CIB under control of *lacI* ^q^/*Ptac*	This work
pSEVA225T	Km^r^, *ori*RK2 *lacZ* promoter probe vector	Silva‐Rocha et al. (2013)
pSEVA225TPaliB	Km^r^, pSEVA225T derivative carrying *P*_*aliB*_::*lacZ* fusion	This work
pET‐28a(+)	Km^r^, *ori*ColE1, *P*_*T7*_, cloning and overexpression vector	Novagen
pET‐28BadR	Km^r^, pET‐28a (+) expressing His_6_‐*badR* under *P*_*T7*_	This work
pK18*mobsacB*	Km^r^, *ori*ColE1, Mob^+^, lacZα. Vector with a *sacB* selection marker for gene replacement by double homologous recombination	Schäfer et al. (1994)
pK18*mobsacB*Δ*badHI*	Km^r^, pK18*mobsacB* containing a chimeric 1.6‐kb *Xba*I/*Hin*dIII fragment carrying the Δ*badHI*	This work
pK18*mobsacB*Δ*AzCIB_1938*	Km^r^, pK18*mobsacB* containing a chimeric 1.4‐kb *Xba*I/*Hin*dIII fragment carrying the Δ*AzCIB_1938(aabC)*	This work

Abbreviations: Gm^r^, gentamicin‐resistant; Km^r^, kanamycin‐resistant; Sm^r^, streptomycin‐resistant.

### Molecular biology techniques

Standard molecular biology techniques were carried out as previously described (Sambrook & Russell, 2001). Plasmid DNA was prepared with a High Pure plasmid isolation kit (Roche Applied Science). DNA fragments were purified with Gene‐Clean Turbo (Q‐BIOgene). Oligonucleotides were supplied by Sigma Co. and their sequences are listed in Supporting Information Table S1. All cloned inserts and DNA fragments were confirmed by DNA sequencing through an ABI Prism 377 automated DNA sequencer (Applied Biosystems Inc.). Transformation of *E*. *coli* cells was carried out by using the RbCl method or by electroporation (Gene Pulser; Bio‐Rad) (Sambrook & Russell, 2001). Plasmids were transferred from *E*. *coli* S17–1λpir (donor strain) into *Azoarcus* and *Paraburkholderia* recipient strains by biparental filter mating as described previously (López‐Barragán et al., 2004). Plasmids were transferred to *Acinetobacter* and *Pseudomonas* strains by electroporation. The protein concentration in cell extracts was determined by the method of Bradford (1976) by using bovine serum albumin as the standard.

### Construction of *Aromatoleum* sp. CIBΔbadHI mutant strain

The *badH* and *badI* genes were deleted by allelic exchange through homologous recombination using the mobilizable plasmid pK18*mobsacB* (Table 1), which allows positive selections of double‐site recombinants using the *sacB* gene of *Bacillus subtilis* (Schäfer et al., 1994). In summary, two DNA flanking regions of the *badH* (719 bp) and *badI* (939 bp) genes were PCR‐amplified with primers Δ*badHI* Fw 1 *Xba*I/Δ*badHI* Rv 1 *Bam*HI and Δ*badHI* Fw 2 *Bam*HI/Δ*badHI* Rv 2 *Hin*dIII (Table S1). Both fragments were digested with *Bam*HI restriction endonuclease, ligated, and the chimeric DNA was then PCR‐amplified with primers Δ*badHI* Fw 1 *Xba*I/Δ*badHI* Rv 2 *Hin*dIII (Table S1). The PCR product was *Xba*I‐*Hin*dIII double‐digested and cloned into the *Xba*I‐*Hin*dIII double‐digested pK18*mobsacB* plasmid. The resulting pK18*mobsacB*Δ*badHI* plasmid (Table 1) was transformed into the *E*. *coli* S17‐1λpir strain (donor strain), and then transferred to *Aromatoleum* sp. CIB (recipient strain) by biparental filter mating (López‐Barragán et al., 2004). Exconjugants containing first site recombination were selected on kanamycin‐containing MC medium harbouring 10 mM glutarate as the sole carbon source for counterselection of donor cells. Second site recombination was selected by growth on the same medium supplemented with 5% sucrose and by plating on glutarate‐containing MC plates supplemented with 5% sucrose. Correct allelic exchange in sucrose‐resistant and kanamycin‐sensitive *Aromatoleum* sp. CIBΔbadHI strain was verified by PCR with the appropriate primers.

### Construction of *Aromatoleum* sp. CIBΔAzCIB_1938 mutant strain

The *AzCIB_1938* gene was deleted by allelic exchange through homologous recombination as indicated above. In summary, two DNA flanking regions (612 and 809 bp) of the *AzCIB_1938* gene were PCR‐amplified with primers Δ*AzCIB_1938* Fw 1 *Xba*I/Δ*AzCIB_1938* Rv 1 *Nde*I and Δ*AzCIB_1938* Fw 2 *Nde*I/Δ*AzCIB_1938* Rv 2 *Hin*dIII (Table S1), *Bam*HI‐digested and ligated. Chimeric DNA was PCR‐amplified with primers Δ*AzCIB_1938* Fw 1 *Xba*I/Δ*AzCIB_1938* Rv 2 *Hin*dIII, *Xba*I‐*Hin*dIII double‐digested, and cloned into the *Xba*I‐*Hin*dIII double‐digested pK18*mobsacB* plasmid. The resulting pK18*mobsacB*Δ*AzCIB_1938* plasmid (Table 1) was transferred from the *E*. *coli* S17‐1λpir strain (donor strain) to *Aromatoleum* sp. CIB (recipient strain) by biparental filter mating, and exconjugants harbouring the first and second recombination events were selected as indicated above. Correct allelic exchange in the sucrose‐resistant and kanamycin‐sensitive *Aromatoleum* sp. CIBΔAzCIB_1938 strain was verified by PCR with the appropriate primers.

### Construction of the pIZBadR, pET‐28BadR, pIZBadRAliA and pSEVA225TPaliB plasmids

For the construction of plasmid pIZBadR (Table 1), the 518‐bp *Xba*I/*Hin*dIII fragment containing the *badR* gene was PCR‐amplified from the genome of strain CIB with primers *badR* Fw *Xba*I/*badR* Rv *Avr*II *Hin*dIII (Table S1), and it was cloned into *Xba*I/*Hin*dIII double‐digested pIZ2 plasmid (Table 1).

The pET‐28BadR plasmid expresses from the *P*_*T7*_ promoter the *badR* gene with a His_6_ tag coding sequence at its 5′‐end. To this end, the *badR* gene was PCR‐amplified with primers *badR* Fw pET *Nde*I/*badR* Rv pET *Hin*dIII (Table S1), and it was cloned into *Nde*I/*Hin*dIII double‐digested pET‐28a plasmid (Table 1).

To construct plasmid pIZBadRAliA (Table 1), which contains the *aliA* gene (encoding the CHC‐CoA ligase from *Aromatoleum* sp. CIB) together with the *badR*, the 1672‐bp *Hin*dIII/*Sac*I fragment containing the *aliA* gene was PCR‐amplified from the genome of strain CIB with primers *aliA* Fw *Hin*dIII/*aliA* Rv *Sac*I (Table S1), and it was cloned into *Hin*dIII/*Sac*I double‐digested pIZBadR plasmid (Table 1).

The pSEVA225T vector (Table 1) was used for the construction of a *PaliB*::*lacZ* translational fusion. The DNA fragment (262 bp) that includes the promoter of *aliB* (*P*_*aliB*_), the leader region and the coding region for the first 17 amino acids of AliB was PCR‐amplified by using primers P*aliB* Fw *Hin*dIII/P*aliB* Rv *Bam*HI (Table S1). The resulting DNA fragment was *Hin*dIII/*Bam*HI double‐digested and cloned upstream of the *lacZ* gene into the double‐digested pSEVA225T promoter probe vector, generating plasmid pSEVA225TPaliB (Table 1).

### Construction of a synthetic *bad‐ali* cassette

A synthetic *bad‐ali* cassette was provided by GenScript Company (New Jersey, USA). In the synthetic cassette the *bad‐ali* genes were present in order aliB*‐aliA*‐badK*‐badH*‐badI*, and they were edited to: (i) remove some restriction enzyme recognition sequences originally present within the structural genes, (ii) add an optimized Shine–Dalgarno sequence to enhance translation, (iii) add *Eco*RI and *Spe*I restriction enzymes sites flanking the cassette for its cloning into the pIZBad plasmid (Table 1). The sequence of the aliB*‐aliA*‐badK*‐badH*‐badI* cassette is provided in Figure S1.

### Construction of a *bad‐ali‐aab* cassette

For the construction of the *bad‐ali‐aab* cassette, we first constructed plasmid pIZBad_A (Table 1). To this end, the 1198‐bp *Spe*I/*Sbf*I fragment containing the *aabA* gene (*AzCIB_1942*) was PCR‐amplified from the genome of strain CIB with primers *AzCIB_1942* Fw *Spe*I/*AzCIB_1942* Rv *Sbf*I (Table S1), and it was cloned into *Spe*I/*Sbf*I double‐digested pIZBad plasmid (Table 1). Then, a 3580‐bp *Sbf*I/*Hin*dIII fragment containing the *aabB*, *aabC* and *aabD* (*AzCIB_1939‐AzCIB_1937*) genes was PCR‐amplified with primers *AzCIB_1939* Fw *Sbf*I/*AzCIB_1937* Rv *Hin*dIII (Table S1), and it was cloned into *Sbf*I/*Hin*dIII double‐digested pIZBad_A plasmid, to generate plasmid pIZBadβ1 (Table 1).

### RNA extraction and RT‐PCR assays

*Aromatoleum* sp. CIB cells were grown aerobically or anaerobically in MC medium containing 3 mM CHC, 3 mM benzoate, or 3 mM pimelate until the culture reached the end of the exponential phase. Cells were harvested and stored at −80°C. Pellets were thawed, and cells were lysed in TE buffer (10 mM Tris–HCl, pH 7.5, 1 mM EDTA) containing 50 mg/ml lysozyme. Total RNA was extracted using High Pure Isolation kit (Roche), and then it was DNase I‐treated according to the manufacturer's instructions (Ambion). The concentration and purity of the RNA samples were assessed using a Nanophotometer Pearl (IMPLEN) according to the manufacturer's protocols. Synthesis of total cDNA was performed by using the Transcriptor First Strand cDNA Synthesis kit (Roche) in 20‐μl reactions containing 1 μg of RNA, 1 mM concentration of each dNTP, 10 units of reverse transcriptase, 20 units of Protector RNase Inhibitor, and 60 μM random hexamers, provided by the manufacturer. The RNA and hexamers were initially heated at 65°C for 10 min and following the addition of the rest of the components, samples were incubated at 25°C for 10 min and then at 55°C for 30 min. Reactions were terminated by incubation at 85°C for 5 min. For the RT‐PCR reactions, cDNA was amplified with 1 unit of AmpliTaq DNA polymerase (Biotools) and 0.5 μM concentrations of the corresponding primer pairs. Control reactions in which reverse transcriptase was omitted from the reaction mixture ensured that DNA products resulted from the amplification of cDNA rather than from DNA contamination.

### β‐Galactosidase assays

The β‐galactosidase activities from the *P*_*aliB*_::*lacZ* reporter fusions were measured with permeabilized cells when cultures reached mid‐exponential phase, as described by Miller (1972).

### Overproduction and purification of His_6_‐BadR

*Escherichia coli* BL21 (DE3) (pET‐28BadR) cells were grown at 37°C in 100 ml of kanamycin‐containing LB medium until the culture reached an OD_600_ of 0.5. Overexpression of the His‐tagged BadR protein was then induced during 5 h by the addition of 0.5 mM IPTG. Cells were harvested at 4°C, resuspended in 10 ml of 20 mM imidazole containing working buffer (50 mM NaH_2_PO_4_, pH 8, 300 mM KCl), and disrupted by passage through a French press operated at a pressure of 20,000 p.s.i. Cell debris was removed by centrifugation at 16,000*g* for 20 min at 4°C, and the resulting supernatant was used as crude cell extract. The His_6_‐BadR protein was purified from the crude cell extract by a single‐step nickel‐chelating chromatography (nickel‐nitrilotriacetic acid spin columns, Qiagen). The column was equilibrated with resuspension buffer, loaded with the crude extract, and washed four times with working buffer plus increasing concentrations of imidazole (20, 75 and 100 mM). The His_6_‐BadR protein was eluted with working buffer containing increasing concentrations of imidazole (0.25, 0.5, 1, 2 and 4 M). The purity of His_6_‐BadR protein was analysed by SDS 12.5% PAGE and subjected to Coomassie staining as described previously (Sambrook & Russell, 2001). When necessary, the protein solutions were dialyzed against working buffer plus 20 mM imidazole, concentrated using Vivaspin 500 columns (Sartorius, 10,000 molecular weight cutoff membrane), and stored at 4°C where they maintained their activity for at least 3 months.

### Gel retardation assays

DNA probe containing the *aliB* promoter (PaliB) and the different DNA mutant probes with substitutions at the BadR‐binding site were PCR‐amplified using specific primers pairs (Table S1) and then digested with *Eco*RI restriction enzyme and single end‐labelled by filling in the overhanging *Eco*RI‐digested ends with [α‐^32^]dATP (6000 Ci/mmol; PerkinElmer Life Sciences) and the Klenow fragment of *E*. *coli* DNA polymerase I as described previously (Sambrook & Russell, 2001). The DNA labelled fragments were purified using GENECLEAN Turbo (Qbiogen). The retardation reaction mixtures contained 20 mM Tris–HCl, pH 7.5, 10% glycerol, 50 mM KCl, 0.05 nM DNA probe, 250 μg/ml bovine serum albumin, 50 μg/ml unspecific salmon sperm DNA, and purified His_6_‐BadR protein in a 9‐μl final volume. After incubation of the retardation mixtures for 20 min at 30°C, mixtures were fractionated by electrophoresis in 5% polyacrylamide gels buffered with 0.5× TBE (45 mM Tris borate, 1 mM EDTA). The gels were dried onto Whatman 3MM paper and exposed to Hyperfilm MP (Amersham Biosciences) accompanied by amplifier screens (Cronex Lightning Plus, DuPont). The radioactivity present in the retardation complexes and free probes was quantified by using a densitometer with the Quantity One software (Bio‐Rad).

## RESULTS AND DISCUSSION

### The *bad‐ali* cluster is responsible for degradation of CHC in *Aromatoleum* sp. CIB

**FIGURE 2:**
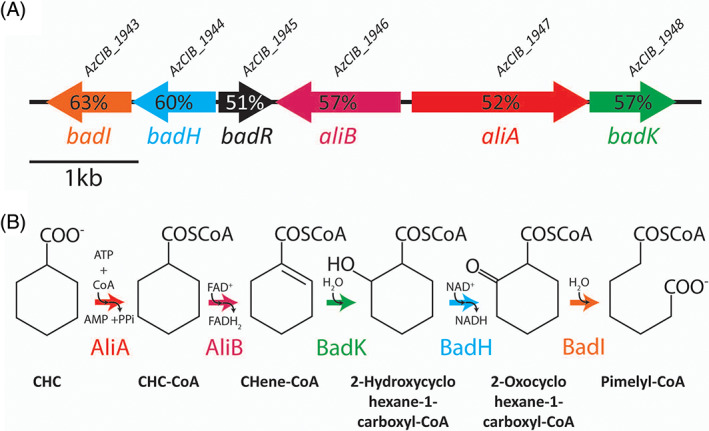
Scheme of the genetic determinants and biochemistry of the bad‐ali pathway for CHC degradation in *Aromatoleum* sp. CIB. (A) Genetic organization of the *bad‐ali* cluster. The colour code of the *ali* and *bad* genes corresponds to that of the functions indicated in panel (B) *badR* (black) encodes a MarR‐type transcriptional regulator. The AzCIB locus tags of the genes are shown at the top; the percentage of amino acid sequence identity to the corresponding proteins in *R*. *palustris* is indicated. B. Biochemistry of the bad‐ali pathway. The enzymes are: AliA, cyclohexane carboxylate CoA‐ligase; AliB, cyclohexanecarboxyl‐CoA dehydrogenase; BadK, CHeneCoA hydratase; BadH, 2‐hydroxycyclohexane‐1‐carboxyl‐CoA dehydrogenase; BadI, 2‐oxocyclohexane‐1‐carboxyl‐CoA hydrolase

**FIGURE 3:**
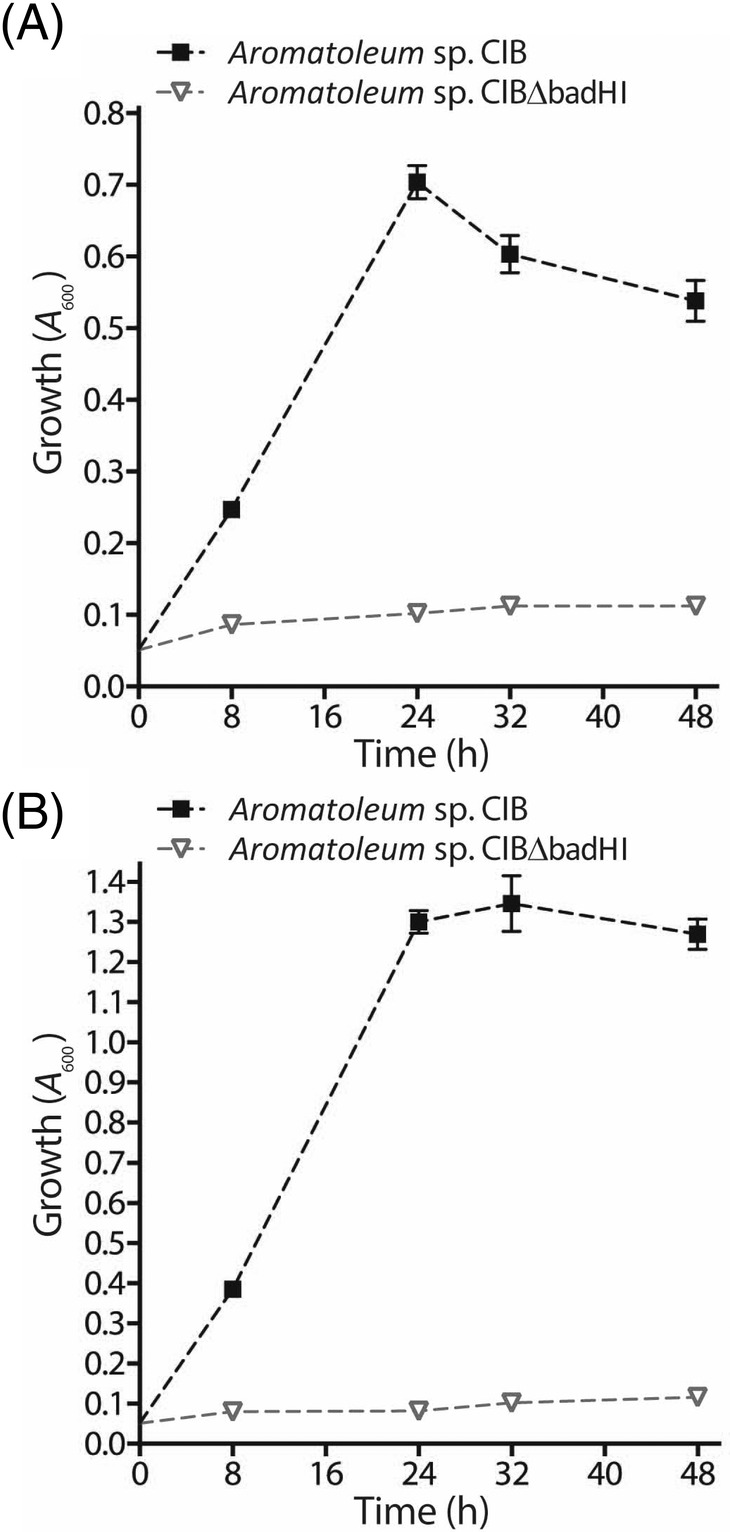
Growth of *Aromatoleum* sp. CIB strains on MC minimal medium containing 3 mM CHC. Symbols: Squares, *Aromatoleum* sp. CIB; triangles, *Aromatoleum* sp. CIBΔbadHI. Growth was monitored by measuring *A*_600_. Values are the mean of three different experiments. Error bars indicate standard deviations. (A) Growth curves under anoxic conditions using 10 mM nitrate as terminal electron acceptor. (B) Growth curves under oxic conditions

An *in silico* analysis of the *Aromatoleum* sp. CIB genome revealed the existence of a gene cluster *(AzCIB_1943‐AzCIB_1948*) whose products showed significant similarity (51%–63% amino acid sequence identity) to the Bad‐Ali proteins from *R*. *palustris*, suggesting that they could be involved in CHC degradation through a biochemical pathway similar to that previously characterized in *R*. *palustris* (Figure 2) (Egland et al., 1997; Pelletier & Harwood, 1998; Pelletier & Harwood, 2000; Martín‐Moldes et al., 2015). The expression of the *bad‐ali* catabolic genes was specifically induced when *Aromatoleum* sp. CIB was grown under anoxic conditions in minimal MC medium with CHC as sole carbon source (Figure S2A), which reinforced the assumption that these genes could be involved in CHC metabolism. To confirm that the *bad‐ali* cluster is the only one involved in CHC degradation in *Aromatoleum* sp. CIB, a *badHI* deletion mutant strain (*Aromatoleum* sp. CIBΔbadHI) was constructed (Table 1). In contrast to the wild‐type CIB strain, the *badHI* mutant strain was unable to grow anaerobically in MC medium with CHC [Figure 3(A)], although it retained its ability to grow with benzoate. Moreover, when the aerobic degradation of CHC was tested, a significant cell growth [Figure 3(B)] and induction of the *bad‐ali* genes (Figure S2B) was observed in the wild‐type *Aromatoleum* sp. CIB, however, no growth was detected with the *badHI* mutant strain [Figure 3(B)]. Thus, these results demonstrated that the *bad‐ali* cluster is the only one involved in CHC metabolism in *Aromatoleum* sp. CIB, ruling out the participation of a CHC pathway as the one reported in strict anaerobes (Kung et al., 2014), and that the bad‐ali pathway is functional under both oxic and anoxic conditions. Moreover, the anaerobic degradation of benzoate, which is carried out by the *bzd* gene products (López‐Barragán et al., 2004), is not influenced by the inactivation of the *bad‐ali* cluster, revealing that aromatic compounds, e.g. benzoate, and alicyclic compounds, e.g. CHC, are metabolized through two different central pathways in strain CIB, which contrasts the previously reported convergence between benzoate and CHC degradation pathways in phototrophs and strict anaerobes (Egland et al., 1997; Kung et al., 2014; Pelletier & Harwood, 2000; Perrotta & Harwood, 1994).

An RT‐PCR‐based transcriptional analysis of the *bad‐ali* cluster from strain CIB demonstrated the CHC‐dependent induction of two divergent transcriptional units, i.e. the *aliA‐badK* and the *aliB‐badH‐badI* operons (Figure S2C), that may be driven by the *P*_*aliA*_ and *P*_*aliB*_ promoters, respectively. The location of the putative *badR* regulatory gene within the *aliB‐badH‐badI* operon but transcribed in the opposite direction [Figure 2(A)] suggests the existence of a regulatory loop that eventually might control the transcriptional and/or post‐transcriptional regulation of *badR*. This peculiar transcriptional organization of the *bad‐ali* cluster in *Aromatoleum* sp. CIB contrasts with that reported in *R*. *palustris* where all the *bad‐ali* catabolic genes form a single operon (*badHbadIaliBaliAbadK*) that is divergently transcribed from the regulatory gene (*badR*) (Egland et al., 1997; Hirakawa et al., 2015).

### The *badR* gene encodes a CHC‐CoA‐dependent transcriptional repressor of the *bad‐ali* catabolic genes

**FIGURE 4:**
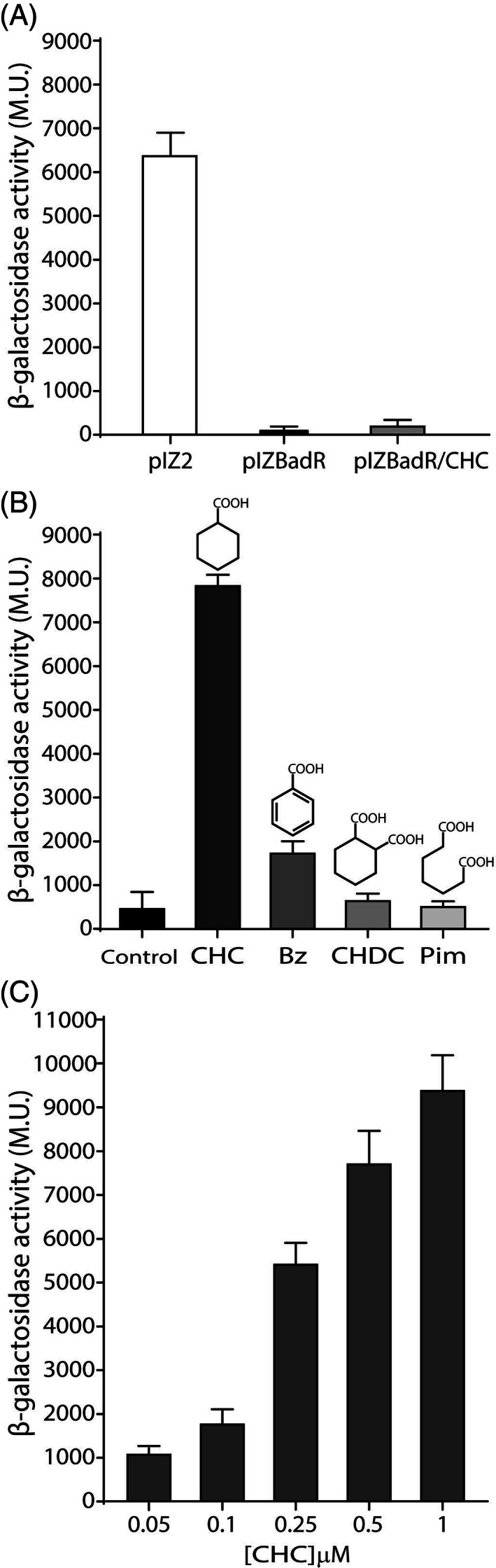
The CHC‐dependent BadR regulator controls the activity of the *P*_*aliB*_ promoter. *Escherichia coli* DH10B cells containing plasmid pSEVA225TPaliB (expresses the *P*_*aliB*_::*lacZ* fusion) and a second plasmid (as indicated below) were grown in LB medium with 1 mM IPTG and different effector molecules (as detailed below). The bars show the β‐galactosidase activity (in Miller units, M.U.) of the cultures determined as indicated under Experimental procedures. Values are the mean of three different experiments. Error bars indicate standard deviations. (A) β‐galactosidase activity of cells grown in LB medium with 1 mM IPTG containing as second plasmid the control plasmid pIZ2 (white bar) or plasmid pIZBadR (black bar). β‐galactosidase activity of *E*. *coli* DH10B cells containing plasmid pIZBadR grown in the presence of 3 mM CHC is also shown (grey bar). (B) β‐galactosidase activity of cells containing plasmid pIZBadRAliA as second plasmid. Cells were grown in the absence (control) or presence of 3 mM CHC, benzoate (Bz), cyclohexane 1,2‐dicarboxylate (CHDC), or pimelate (Pim). (C) β‐galactosidase activity of cells containing plasmid pIZBadRAliA as second plasmid and grown in the presence of increasing concentrations (from 0.05 to 1 μM) of CHC

In *R*. *palustris* the *bad‐ali* genes are under control of a MarR‐type transcriptional repressor, the BadR protein. The BadR binding site (CAATacATTG) at the target *badH* promoter includes an inverted repeat sequence (underlined) separated by a 2‐bp spacer (lowercase) (Hirakawa et al., 2015). Interestingly, two similar BadR operator regions could be identified in the *aliB‐aliA* intergenic region of strain CIB. These potential BadR operators overlap the predicted −35 boxes of the divergent *P*_*aliB*_ and *P*_*aliA*_ promoters (Figure S3), suggesting that BadR could behave as a transcriptional repressor of the *bad‐ali* catabolic genes in *Aromatoleum* sp. CIB. To confirm the repressor role of BadR, the *badR* gene was cloned under control of the *lacI* ^*q*^ */P*_*tac*_ regulatory couple in plasmid pIZBadR, and a *P*_*aliB*_::*lacZ* translational fusion was constructed in plasmid pSEVA225TPaliB (Table 1). *Escherichia coli* DH10B strain carrying *P*_*aliB*:_:*lacZ* showed a significant β‐galactosidase activity, indicating that the *P*_*aliB*_ promoter was functional in this heterologous host [Figure 4(A)]. However, the expression of the *badR* gene in the *E*. *coli* DH10B strain carrying *P*_*aliB*_::*lacZ* led to a lack of β‐galactosidase activity indicating inhibition of the *P*_*aliB*_ promoter [Figure 4(A)]. Therefore, these results are in agreement with the previous hypothesis that BadR acts as a transcriptional repressor of the *bad‐ali* catabolic genes, likely by blocking access of the RNA polymerase to the target promoter as suggested also in the *bad‐ali* cluster of *R*. *palustris* (Hirakawa et al., 2015).

In order to identify the BadR effector molecule that induces the expression of the *bad‐ali* genes, β‐galactosidase assays were performed in *E*. *coli* DH10B strain carrying plasmids pSEVA225TPaliB (*P*_*aliB*_::*lacZ*) and pIZBadR (*badR*) and grown in the presence of CHC. There was no expression of the reporter fusion in presence of CHC [Figure 4(A)], hence suggesting that the effector molecule is not the initial substrate but rather some further CoA derivative intermediate formed during CHC degradation. To prove this hypothesis, the *aliA* gene, predicted to encode a CHC CoA ligase that generates the first pathway intermediate (CHC‐CoA) (Figure 2), was cloned in plasmid pIZBadR generating plasmid pIZBadRAliA (Table 1) that expresses both the *badR* and *aliA* genes under control of the *lacI* ^*q*^ */P*_*tac*_ regulatory couple. *Escherichia coli* DH10B (pSEVA225TPaliB, pIZBadRAliA) showed a clear activation of the *P*_*aliB*_ promoter only in presence of CHC [Figure 4(B)], suggesting that AliA is indeed a CoA ligase that activates CHC to CHC‐CoA, and this molecule acts as the effector that binds to BadR inducing the expression of the *bad‐ali* genes. Furthermore, when CHC was replaced by its aromatic analogue, i.e. benzoate, a minor activation of *P*_*aliB*_ was observed [Figure 4(B)], and no significant induction was obtained in presence of di‐carboxylic substrates either alicyclic, e.g. cyclohexane 1,2‐dicarboxylate, or aliphatic, e.g. pimelate [Figure 4(B)]. Nevertheless, the minor activation of *P*_*aliB*_ observed in *E*. *coli* in the presence of benzoate does not appear to have a physiological relevance in *Aromatoleum* sp. CIB since the *bad‐ali* genes were not induced when strain CIB grew with benzoate (Figure S2). All these results suggest that the AliA/BadR regulatory system is specific of CHC.

Cells harbouring the *aliA/badR/PaliB*::*lacZ* genetic system described in this work constitute the first CHC whole‐cell biosensor reported so far. To determine the range of CHC concentrations that switched‐on the expression of the reporter *lacZ* gene we monitored β‐galactosidase activity in *E*. *coli* DH10B (pSEVA225TPaliB, pIZBadRAliA) cells grown in the presence of increasing concentrations of CHC. A dose–response that spans from 0.1 μM CHC (2‐fold induction) to 1 μM CHC (10‐fold induction) was observed [Figure 4(C)].

So far, the transcriptional regulation of the *bad‐ali* genes had been only studied in *R*. *palustris*. The 2‐oxocyclohexane‐1‐carboxyl‐CoA, the fourth intermediate in the bad‐ali pathway (Figure 2), was shown to interact with BadR and induce expression of the *bad‐ali* genes (Hirakawa et al., 2015). In this work it has been demonstrated that the first intermediate of CHC degradation, i.e. CHC‐CoA, interacts with BadR from *Aromatoleum* sp. CIB and abrogates repression of the *P*_*aliB*_ promoter; however, it cannot be discarded that other CoA derivatives of the CHC degradation pathway could also behave as inducers. Whether the orthologous BadR regulators from *R*. *palustris* and *Aromatoleum* sp. CIB have different effector specificity or, on the contrary, they recognize the same range of inducer(s), should be subject of further studies.

### In vitro DNA binding assays and characterization of the BadR operator site

**FIGURE 5:**
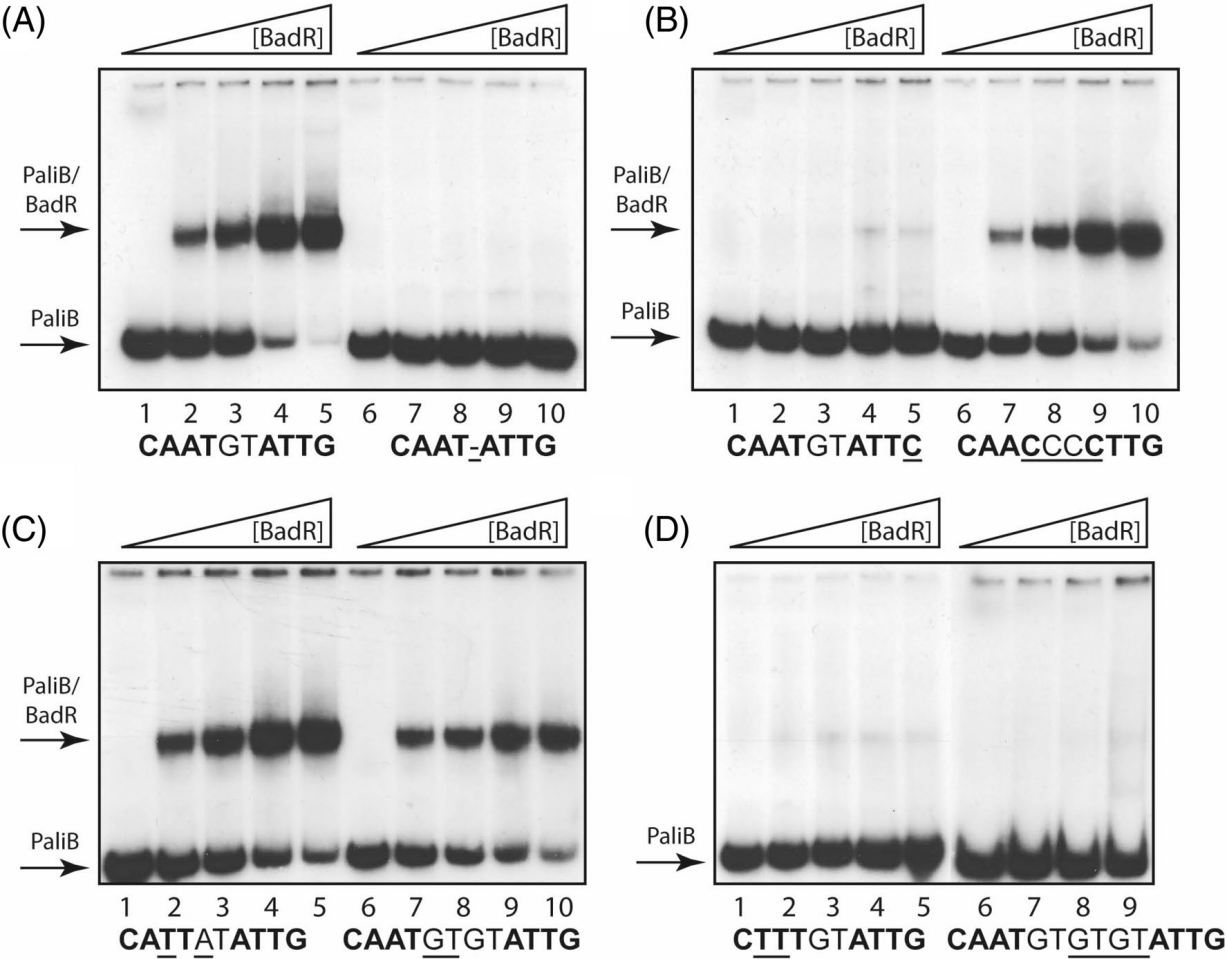
Characterization of the BadR operator region in the *P*_*aliB*_ promoter. Gel retardation assays were performed as detailed in Experimental procedures by using increasing concentrations of purified His_6_‐BadR protein (lanes 1 and 6, 0 pmol; lanes 2 and 7, 1 pmol; lanes 3 and 8, 2 pmol; lanes 4 and 9, 4 pmol; lanes 5 and 10, 8 pmol) and the wild‐type PaliB probe (panel A, lanes 1–5), a mutant PaliB probe without the 2‐bp spacer in the BadR‐binding site (panel A, lanes 6–10), or mutant PaliB probes with nucleotide substitution(s) in the BadR operator region (panels B–D). The nucleotide sequence of the different BadR operator regions is detailed at the bottom of the panels. The 4‐bp inverted repeats and the 2‐bp spacer are indicated in bold and plain text, respectively. Nucleotide substitutions are underlined. The P_aliB_ probes and BadR/*P*_*aliB*_ complexes are marked with arrows

To confirm the direct and specific binding of BadR to its target *P*_*aliB*_ promoter, in vitro DNA binding assays were accomplished. To this end, the BadR regulator was overproduced in *E*. *coli* and purified by nickel affinity chromatography as a soluble N‐terminal His_6_‐tagged protein (see the Experimental procedures). To demonstrate that the purified BadR regulatory protein directly interacted with the *P*_*aliB*_ promoter, gel retardation assays were performed using as probe a P^32^‐labelled 100‐bp DNA fragment that contained the *aliB* promoter region (PaliB probe). Purified His_6_‐BadR was able to retard the migration of the PaliB probe in a protein concentration‐dependent manner [Figure 5(A), lanes 1–5], in agreement with the observed in vivo inhibition of *P*_*aliB*_ by the *badR* gene product [Figure 4(A)].

As indicated above, a putative 10‐bp BadR operator region (CAATgtATTG) is present in the *P*_*aliB*_ promoter from *Aromatoleum* sp. CIB (Figure S3). To confirm that this palindromic region constitutes the BadR recognition site in strain CIB and to study the role of the 4‐bp inverted repeat sequence and that of the 2‐bp spacer, gel retardation assays have been performed with DNA probes harbouring different nucleotide substitutions at the predicted operator region. Thus, when the palindrome was disrupted by substitution of the first or second nucleotide of the inverted repeat sequence, the BadR protein was unable to bind to the target DNA [Figure 5(B), lanes 1–5; Figure 5(D), lanes 1–5]. However, substitutions at the third or fourth nucleotide of the inverted repeat sequence did not cause a significant reduction of BadR binding [Figure 5(B), lanes 6–10; Figure 5(C), lanes 1–5]. On the other hand, if the 2‐bp spacer was removed [Figure 5(A), lanes 6–10] or extended to 6‐bp [Figure 5(D), lanes 6–9], the mutated probes could not interact with BadR. On the contrary, the extension of the spacer region to 4‐bp was not preventing BadR binding [Figure 5(C), lanes 6–10]. All these data taken together suggest that **CA**ATnnAT**TG** is the sequence of the BadR operator, where the two first nucleotides (in bold) of the conserved 4‐bp inverted repeat sequence appear to be essential for BadR binding, and the length of the spacer region should not be lower than 2‐bp and higher than 4‐bp to maintain the inverted repeat sequences at the same face of the DNA helix.

### Engineering a synthetic *bad‐ali* cassette for pimelyl‐CoA production

To demonstrate that the *aliA*, *aliB*, *badK*, *badH* and *badI* genes from *Aromatoleum* sp. CIB are the only ones needed for the conversion of CHC into pimelyl‐CoA, we engineered a synthetic *bad‐ali* cassette (aliB*‐aliA*‐badK*‐badH*‐badI*). The modularity of the *bad‐ali* cassette was facilitated by removing some restriction enzyme sites present in the native *ali‐bad* genes and by adding additional sites that allowed the genetic manipulation of the cassette. The synthetic *bad‐ali* cassette was expressed under control of the *lacI* ^*q*^ */P*_*tac*_ regulatory couple and harbouring optimized Shine–Dalgarno sequences, and it was cloned into a broad‐host‐range vector generating plasmid pIZBad (Table 1). When plasmid pIZBad was transferred to *E*. *coli* JW3375‐1, an *E*. *coli* Δ*bioH* mutant strain auxotrophic for the vitamin biotin since it cannot synthesize the pimelyl‐CoA precursor, the resulting strain was able to grow in a minimal medium lacking biotin if CHC was added (Figure S4), hence indicating that the *bad‐ali* cluster from strain CIB was able to supply the pimelyl‐CoA needed for biotin synthesis (Bernstein et al., 2008). Thus, this result confirms that the synthetic *bad‐ali* cassette from *Aromatoleum* sp. CIB is functional and allows conversion of CHC into pimelyl‐CoA. The use of this synthetic cassette in bacteria that overproduce biotin, e.g. *Pseudomonas mutabilis* ATCC31014 (Xiao et al., 2019), could be further explored as a genetic strategy to increase biotin production when feeding the recombinant biocatalysts with CHC as source of pimelyl‐CoA (Bernstein et al., 2008).

### The *bad‐ali* cluster is widely distributed in bacteria

**FIGURE 6:**
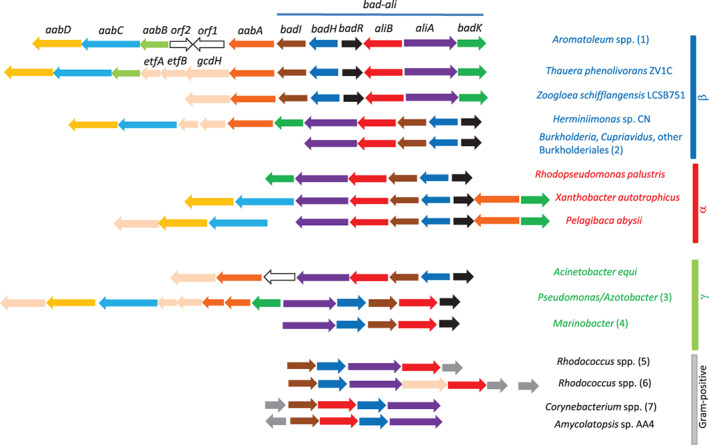
Comparison of the *bad‐ali* clusters from different bacteria. The *bad‐ali* genes and their neighbouring *aab* genes are shown by colour arrows. Black and grey arrows represent the MarR‐type and TetR‐type transcriptional regulators, respectively. White arrows are genes of unknown function. Light brown arrows represent the *gcdH* gene and associated *etfAB* genes. β‐, α‐ and γ‐proteobacterial strains are indicated with blue, red and green letters, respectively. Gram‐positive strains are indicated in grey. Numbers correspond to the following strains/genera: (1) *Aromatoleum* sp. CIB, *Aromatoleum* sp. DN11, *A*. *toluclasticus* ATCC70060, *A*. *tolulyticus* ATCC51758, *Azoarcus* sp. KH32C, *A*. *aromaticum* EbN1 (in this strain the *aabBCD* genes are not linked to the *bad‐aabA* genes), *Aromatoleum* sp. PA01, *A*. *petrolei* ToN1. (2) *Paraburkholderia*, *Ralstonia*, *Comamonas*, *Pandoraea*, *Polaromonas*, *Achromobacter*, *Acidovorax*, *Variovorax*, *Leptothrix*, *Aquabacterium*, *Alicycliphilus*. (3) *Pseudomonas silesiensis*, *P*. *fluorescens*, *P*. *sagittaria*, *Pseudomonas* sp. TCU‐HL1, *Azotobacter beijerinckii*. (4) *Marinobacter adhaerens*, *M*. *salinus*. (5) *Rhodococcus rhodochrous* DSM43241, *R*. *pyridinivorans* KG‐16, *R*. *jostii* RHA1, *R*. *opacus* R7, *R*. *opacus* PD630. (6) *Rhodococcus aetherivorans* BCP1, *R*. *ruber* BKS 20‐38. (7) *Corynebacterium terpenotabidum*, *C*. *efficiens*

As indicated in the Introduction, the *bad‐ali* cluster had been initially studied in an anaerobic degrader of aromatic compounds, i.e. *R*. *palustris* (Egland et al., 1997; Pelletier & Harwood, 2000; Perrotta & Harwood, 1994). Here we show that the *bad‐ali* cluster from another anaerobic degrader of aromatics, i.e. the denitrifying *Aromatoleum* sp. CIB strain is also responsible for CHC degradation to pimelyl‐CoA. Putative *bad‐ali* clusters involved in CHC degradation have been also predicted in the genomes of other anaerobic degraders of aromatics such as *A*. *aromaticum* EbN1, *A*. *petrolei* ToN1 and *Herminiimonas* sp. CN strains (Kim et al., 2014; Weiten et al., 2021). Nevertheless, *bad‐ali* genes predicted to be involved in CHC degradation have been also identified in *Cupriavidus gilardii* CR3 (Wang et al., 2015), *Rhodococcus aetherivorans* (Presentato et al., 2018) and *Rhodococcus opacus* R7 (Zampolli et al., 2020), three strains unable to degrade aromatic compounds under anoxic conditions, hence revealing that the *bad‐ali* cluster is not always associated with bacteria able to anaerobically degrade aromatic compounds. To explore in more detail the distribution and organization of the *bad‐ali* cluster in microorganisms, we performed an *in silico* search within the microbial genomes databases. As shown in Figure 6, the *bad‐ali* cluster is widely distributed among bacteria, revealing that the bad‐ali pathway is more widespread than previously thought. Whereas *bad‐ali* clusters from Gram‐negative bacteria contain a *badR* regulatory gene encoding a MarR‐type regulator that is usually divergently transcribed to the *bad* catabolic genes, *bad‐ali* clusters from Gram‐positive bacteria harbour a TetR‐like regulatory gene that is usually transcribed in the same direction as the other *bad‐ali* genes. Interestingly, in most Gram‐negative bacteria a consensus BadR binding site (CAAN_4_TTG) can be identified in the intergenic region of the two divergent operons, which suggests that the transcriptional regulation of the *bad‐ali* genes is conserved in the majority of these *bad‐ali* clusters.

Within β‐proteobacteria, the genetic organization of the *bad‐ali* cluster in members of the Rhodocyclales group, i.e. *Aromatoleum*, *Thauera* and *Zoogloea* strains, is clearly different than that in members of the Burkholderiales group, i.e. *Herminiimonas*, *Burkholderia*, *Paraburkholderia*, *Cupriavidus*, *Ralstonia*, *Comamonas*, *Pandoraea*, *Polaromonas*, *Achromobacter*, *Acidovorax*, *Variovorax*, *Leptothrix*, *Aquabacterium*, *Alicycliphilus*, and so on (Figure 6). Moreover, in most strains of the Burkholderiales group their *bad‐ali* clusters lacked a *badK* orthologue (Figure 6). The absence of a *badK* orthologue was also observed in other *bad‐ali* clusters from Gram‐negative and Gram‐positive bacteria (Figure 6), although in most cases a *badK*‐like gene could be found in another genomic location. An additional *badKHI* cluster lacking the *aliAB* and *badR* genes has been also described in the genomes of *A*. *petrolei* ToN1 and *A*. *aromaticum* pCyN1, and it was suggested to be involved in monoterpenes degradation (Weiten et al., 2021).

### Identification of the *aab* cluster responsible of the lower pathway for degradation of CHC and benzoate in *Aromatoleum* sp. CIB

**FIGURE 7:**
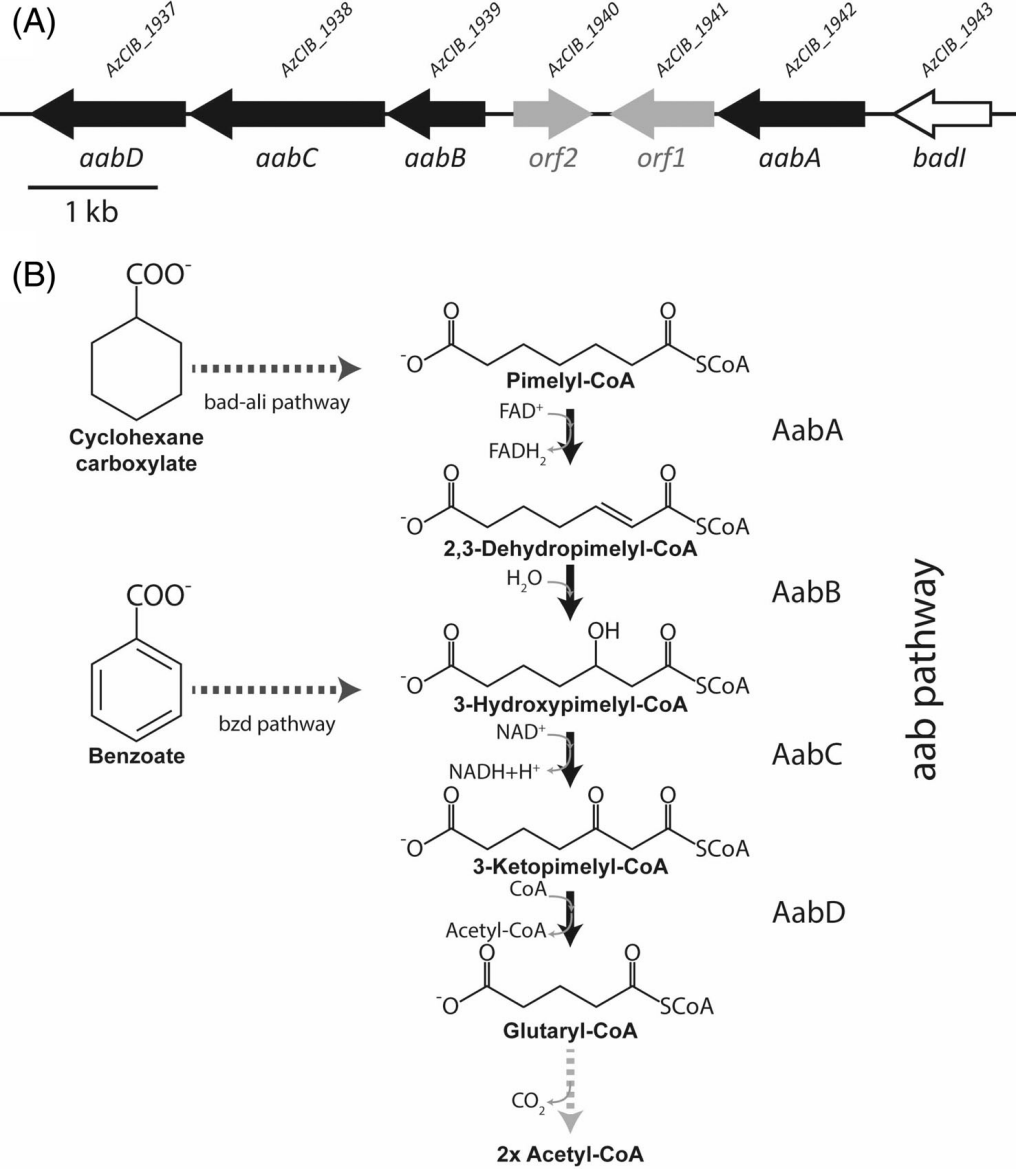
The bad‐ali and bzd pathways converge at the aab pathway in *Aromatoleum* sp. CIB: scheme of the genes and enzyme reactions involved in the aab pathway. (A) Genetic organization of the *abb* cluster. The function of the *aabA*, *aabB*, *aabC* and *aabD* genes (black) is indicated in panel B. The AzCIB locus tags of the genes are shown at the top. *orf1* and *orf2* are two genes (grey) of unknown function. The *badI* gene (white) is also shown. (B) The CHC degradation (bad‐ali pathway) and benzoate degradation (bzd pathway) generate pimelyl‐CoA and 3‐hydroxypimelyl‐CoA, respectively, two intermediates of the aab pathway. Metabolites and enzymatic reactions of the aab pathway are shown. The enzymes are: AabA, pimelyl‐CoA dehydrogenase; AabB, 2,3‐dehydropimelyl‐CoA hydratase; AabC, 3‐hydroxypimelyl‐CoA dehydrogenase; AabD, 3‐oxopimelyl‐CoA thiolase. Glutaryl‐CoA is further metabolized to acetyl‐CoA through a glutaryl‐CoA dehydrogenase and a set of short‐chain fatty acids β‐oxidation reactions

*Aromatoleum* sp. CIB degrades benzoate under anoxic conditions via the central bzd pathway generating 3‐hydroxypimelyl‐CoA (Carmona et al., 2009; Durante‐Rodríguez et al., 2018; Martín‐Moldes et al., 2015). As we have shown above, CHC degradation in strain CIB involves a different central pathway coded by the *bad‐ali* cluster that generates pimelyl‐CoA as final product (Figure 2). Thus, the bzd and bad‐ali pathways should converge in a common lower pathway that funnels both pimelyl‐CoA and 3‐hydroxypimelyl‐CoA into the central metabolism of *Aromatoleum* sp. CIB. In this sense, an *in silico* search in the CIB genome allowed us to identify a cluster, hereafter referred to as *aab* cluster (aromatic alicyclic beta‐oxidation), located next to the *bad‐ali* cluster and containing genes, i.e. *aabA*, *aabB*, *aabC* and *aabD*, whose products show a significant similarity to enzymes involved in a β‐oxidation pathway of aliphatic dicarboxylic acids (Harrison & Harwood, 2005; López‐Sánchez et al., 2010; Parke et al., 2001) (Figure 7). Genes *AzCIB_1940* (*orf2*) and *AzCIB_1941* (*orf1*) encode a putative thioesterase (PaaY‐like) and reductase, respectively, whose function in the predicted β‐oxidation pathway is still unknown. To check whether the *aab* cluster becomes induced when strain CIB grows anaerobically on C_7_ acids either aromatic, e.g. benzoate, alicyclic, e.g. CHC, or aliphatic, e.g. pimelate, we performed gene expression studies. The *aabD* gene was induced during growth in CHC and benzoate, but not with pimelate (Figure S5A). Thus, these results suggested that cluster *aab* is involved in the lower pathway for CHC and benzoate catabolism but not for pimelate degradation. Interestingly, whereas the *aabA* gene becomes highly induced when the cells grow with CHC, there was no significant induction when using benzoate (or pimelate) as carbon sources (Figure S5B). This result is in agreement with the fact that metabolism of CHC (aerobic and anaerobic) generates pimelyl‐CoA; however, anaerobic benzoate degradation produces 3‐hydroxypimelyl‐CoA whose catabolism does not require the AabA enzyme (Figure 7). The location of the *aabA* gene just downstream of *badI* [Figure 7(A)] and the specific induction of both genes when cells grew with CHC led us to check whether these two genes were indeed co‐transcribed as part of the same operon. RT‐PCR amplification of the *badI‐aabA* intergenic region in RNA samples from cells grown with CHC confirmed that these two genes were co‐transcribed (Figure S2C), thus indicating that *aabA* is indeed the last gene of the *aliB‐badH‐badI‐aabA* operon. No induction of the *orf1* and *orf2* genes was observed when cells were grown with CHC or benzoate. The location of the *aabA* gene within the *bad‐ali* cluster in *Aromatoleum* sp. CIB was not observed previously in the *bad‐ali* cluster from *R*. *palustris* but appears to be a general feature among *bad*‐*ali* clusters from members of the Rhodocyclales group, i.e. *Aromatoleum Thauera* and *Zoogloea* strains, as well as in *bad‐ali* clusters of several β‐, α‐ and γ‐proteobacteria (Figure 6). This genetic organization may reflect regulatory issues and could assure an adequate expression level of the *aabA* gene when cells feed on CHC (see below).

**FIGURE 8:**
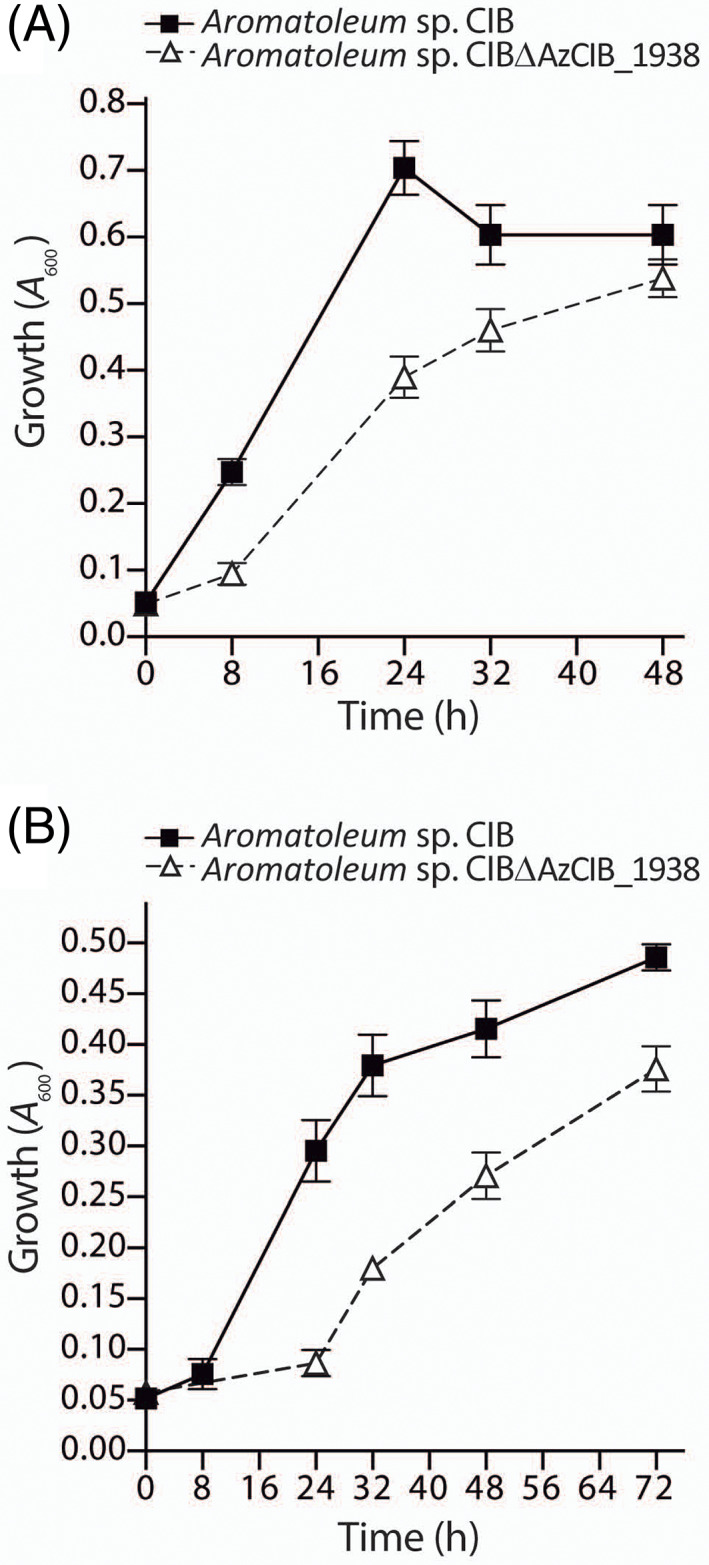
Anaerobic growth of *Aromatoleum* sp. CIB strains in MC minimal medium. Symbols: squares, *Aromatoleum* sp. CIB; triangles, *Aromatoleum* sp. CIBΔAzCIB_1938. Growth was monitored by measuring *A*_600_. Values are the mean of three different experiments. Error bars indicate standard deviations. (A) Growth curves with 3 mM CHC. (B) Growth curves with 3 mM benzoate

To confirm whether the *aabBCD* operon was essential for the anaerobic degradation of CHC and benzoate in *Aromatoleum* sp. CIB, we constructed a mutant strain harbouring a disrupted *aabC* gene. Although the resulting strain, *Aromatoleum* sp. CIBΔAzCIB_1938 (Table 1), was able to use benzoate and CHC as sole carbon sources, it showed a significantly longer lag phase than the wild‐type CIB strain (Figure 8). These results suggested that the *aabBCD* operon was indeed involved in the lower pathway for benzoate/CHC degradation but, when inactivated, it could be replaced by additional β‐oxidation functions induced in the adapted mutant cells. In this sense, a second β‐oxidation cluster (AzCIB_2912‐2917) is also present in the CIB genome. We have observed an induction of the *aabC* paralogue (AzCIB_2912) when *Aromatoleum* sp. CIBΔAzCIB_1938 was grown in CHC or benzoate (Figure S5C), suggesting that cluster AzCIB_2912‐2917, which is not significantly expressed in the wild‐type strain growing with benzoate or CHC, becomes induced and replaces the *aab* cluster when the latter is not functional. The existence of multiple clusters encoding putative dicarboxylic acid β‐oxidation pathways appears to be a common feature within bacterial genomes (Butler et al., 2007; Carmona et al., 2009; Harrison & Harwood, 2005; López‐Sánchez et al., 2010; Rabus et al., 2005) and it might represent an adaptive advantage when the occasional failure of one pathway is backed by the induction of an equivalent pathway.

In most *Aromatoleum* strains the *aab* genes are located adjacent to the *bad‐ali* cluster, as shown above with *Aromatoleum* sp. CIB (Figure 6). However, in *A*. *aromaticum* EbN1 the *aabBCD* genes are not adjacent to the *bad‐ali* cluster (that however includes the *aabA* gene) but close to the *bss‐bbs* genes for anaerobic degradation of toluene (Kühner et al., 2005; Rabus et al., 2005). Since *aab‐bad‐ali* genes form a supraoperonic clustering in most *Aromatoleum* strains, it is tempting to speculate that in these denitrifying bacteria the *aab* genes have initially evolved for CHC degradation but then they were recruited also for the lower pathway of anaerobic benzoate degradation. In *Thauera phenolivorans* the *bad‐ali* genes are not only associated to the *aab* genes but to a *gcdH* and *etfAB* genes likely involved in the degradation of the glutaryl‐CoA generated by the *aab‐ali* gene products (Blázquez et al., 2008; Durante‐Rodríguez et al., 2018; Estelmann & Boll, 2014; Husain & Steenkamp, 1985). The association of *gcdH* to the *aab‐bad‐ali* genes can be also observed in other Gram‐negative bacteria (Figure 6).

### Engineering a synthetic *bad‐ali‐aab* catabolic module to expand CHC degradation in heterologous hosts

**FIGURE 9:**
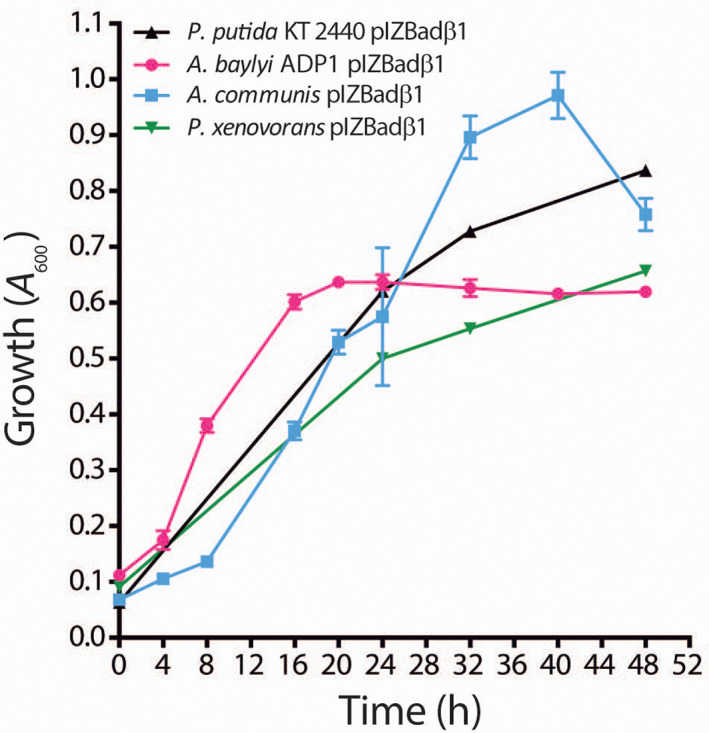
Growth with CHC of different environmentally relevant bacteria harbouring the *bad‐ali‐aab* recombinant cassette. *Acinetobacter baylyi* ADP1 (red), *Azoarcus communis* SWub3 (blue), *Pseudomonas putida* KT2440 (black) and *Paraburkholderia xenovorans* LB400 (green) containing plasmid pIZBadβ1 that expresses the *bad‐ali‐aabA‐aabBCD* cassette were grown aerobically in MC minimal medium containing 3 mM CHC. Growth was monitored by measuring *A*_600_. Values are the mean of three different experiments. Error bars indicate standard deviations

As we have shown above, the synthetic *bad‐ali* cassette confers the ability to produce pimelyl‐CoA when *E*. *coli* is growing in the presence of CHC (Figure S4). Therefore, we checked whether this synthetic cassette allows growth with CHC of bacteria harbouring an endogenous *aab* cluster for degradation of pimelyl‐CoA, as in *Acinetobacter baylyi* ADP1 (Parke et al., 2001), or 3‐hydroxypimelyl‐CoA, as in *Azoarcus communis* SWub3 (Zamarro et al., 2017). However, recombinant *A*. *baylyi* ADP1 and *A*. *communis* SWub3 strains harbouring plasmid pIZBad (*bad‐ali* cassette) were unable to use CHC as sole carbon and energy source, suggesting that expression of endogenous *aab* genes in these bacterial hosts is not induced by the conversion of CHC into pimelyl‐CoA. Since the *bad‐ali* operon from *Aromatoleum* sp. CIB includes also the *aabA* gene and this extended *bad‐ali* cluster is conserved in many bacteria (Figure 6), the co‐expression of *aabA* with the rest of *bad‐ali* genes might be essential for the efficient mineralization of the pimelyl‐CoA generated during CHC degradation. To try to overcome these putative gene expression problems, a complete *aab* cassette harbouring genes *aabA* and *aabBCD* from strain CIB with optimized Shine–Dalgarno sequences was engineered and combined with the synthetic *bad‐ali* cassette generating plasmid pIZBadβ1 (Table 1). When the broad‐host‐range plasmid pIZBadβ1 was introduced into *A*. *baylyi* ADP1 and *A*. *communis* SWub3, the resulting recombinant strains grew with CHC (Figure 9), indicating that the controlled expression of the complete set of *bad‐ali* and *aab* genes was required for the efficient catabolism of CHC in these heterologous hosts. Moreover, we tested whether pIZBadβ1 could confer the ability to degrade CHC to bacterial strains, such as *Paraburkholderia xenovorans* LB400 or *P*. *putida* KT2440 (Table 1), that are unable to use pimelyl‐CoA although they can metabolize glutaryl‐CoA [final product of the aab pathway, Figure 7(B)]. Interestingly, the two recombinant strains acquired the ability to grow in minimal medium with CHC as only carbon source (Figure 9). These results confirmed that the *aab* genes were responsible for pimelyl‐CoA degradation, and revealed for the first time that the *bad‐ali‐aab* cassette could be used as an efficient genetic tool to expand the catabolic abilities of a wide variety of bacteria for CHC metabolism. Genetically amenable and industrially relevant bacteria, e.g. *A*. *baylyi*, *P*. *putida*, *P*. *xenovorans* (Biggs et al., 2020; Nikel & de Lorenzo, 2018; Pérez‐Pantoja et al., 2012; Weimer et al., 2020), able to degrade CHC can be of interest in bioremediation and, eventually, in the conversion of CHC into bioplastics, e.g. polyhydroxybutyrate, mcl‐polyhydroxyalkanoates (Salvachúa et al., 2020; Urtuvia et al., 2018; Wierckx et al., 2015) and other bio‐products (Santala et al., 2021). On the other hand, expression of the *bad*‐*ali* and *bad‐ali‐abb* cassettes into suitable bacterial hosts unable to catabolize dicarboxylic acids could be also of interest for bioconversion of CHC into monomers, e.g. pimelic and glutaric acids, that can be used for production of bio‐based polymers (Chung et al., 2015; Turk et al., 2016).

## CONCLUSIONS

In this work it was demonstrated that the CHC central degradation pathway (bad‐ali pathway) and the anaerobic benzoate central degradation pathway (bzd pathway) do not share common intermediates but rather they represent independent pathways in denitrifying bacteria such as *Aromatoleum* sp. CIB. This strategy for the anaerobic catabolism of benzoate and CHC contrasts with the previous assumption that aromatic and alicyclic compounds are degraded to a characteristic joint intermediate that can be CHeneCoA in phototrophs (*R*. *palustris*) or CHdieneCoA in all other CHC‐degrading anaerobic bacteria (Kung et al., 2014) (Figure 1). In *Aromatoleum* sp. CIB, and likely in other facultative anaerobes that degrade both CHC and benzoate, the bad‐ali pathway and the anaerobic bzd pathway generate pimelyl‐CoA and 3‐hydroxypimelyl‐CoA, respectively, that are metabolized via a common aab pathway usually encoded in the close vicinity to the *bad‐ali* genes forming a supraoperonic clustering. The *bad‐ali* cluster was shown to be more widespread than previously thought, and it was identified in the genome of a wide variety of Gram‐negative and Gram‐positive bacteria, many of which are aerobic bacteria that do not degrade aromatic compounds under anoxic conditions. Accordingly, the *bad‐ali* cluster was shown to be also responsible of aerobic CHC degradation in *Aromatoleum* sp. CIB, confirming that it encodes an oxygen‐independent pathway as in *R*. *palustris* (Hirakawa et al., 2015; Küver et al., 1995).

The transcriptional organization of the *bad‐ali* cluster from *Aromatoleum* sp. CIB, i.e. two divergent catabolic promoters, was shown to be different to that previously reported in *R*. *palustris* (Egland et al., 1997; Hirakawa et al., 2015), and the BadR transcriptional repressor (MarR‐type) was shown to recognize CHC‐CoA, the first intermediate of the pathway, as effector. A consensus sequence CAAnnnnTTG was characterized as the BadR operator in strain CIB, and this operator is generally conserved in the target promoters of *bad‐ali* clusters from Gram‐negative bacteria, hence suggesting that the transcriptional regulation of the *bad‐ali* genes by a BadR repressor is also conserved in all these bacteria.

A previously unreported association of the *bad‐ali* genes with the *aab* genes that convert pimelyl‐CoA into glutaryl‐CoA has been elucidated. This supraoperonic clustering appears to be conserved in most members of the Rhodocyclales group (β‐proteobacteria) as well as in some α‐ and γ‐proteobacteria. A synthetic *bad‐ali‐aab* catabolic module was engineered under control of well‐known regulatory signals, and it was shown to confer CHC degradation abilities to a variety of heterologous hosts. This *bad‐ali‐aab* catabolic module constitutes an unprecedented genetic tool that could be used to design efficient biocatalysts to remove and valorize CHC towards the production of bio‐products of industrial interest, e.g. bioplastics and bio‐monomers for the synthesis of bio‐based polymers, for a circular and sustainable economy.

## CONFLICT OF INTEREST

The authors declare that there is no conflict of interest.

## Supporting information


**FIGURE S1** Nucleotide sequence of the synthetic *bad‐ali* cassette. The edited sequence of the aliB*(pink), aliA* (red), badK* (green), badH* (blue) and badI* (orange) genes is shown. The optimized Shine‐Dalgarno sequences with stop codons in all three reading frames and some additional restriction sites (*Eco*RI and *Spe*I) flanking the cassette are indicated in black.

**FIGURE S2** Analysis of the expression of the *bad‐ali* genes in *Aromatoleum* sp. CIB. Total RNA was isolated from cells grown in MC medium containing 3 mM benzoate (Bz) or 3 mM CHC as sole carbon source until the end of the exponential phase. The expression of the target genes and intergenic regions was monitored by RT‐PCR using the oligonucleotide pairs detailed in Table S1. Agarose gel electrophoresis of RT‐PCR products is shown. Lane M, molecular size markers (Quick‐Load™ 100 bp DNA Ladder from New England BioLabs). Lanes Bz and CHC indicate RT‐PCR reactions from RNA isolated of cells grown with Bz and CHC, respectively.
A. Expression of *badI*, *badH*, *aliB*, *aliA* and *badK* genes from cells grown under anoxic conditions.
B. Expression of *badI*, *badH*, *aliB*, *aliA* and *badK* genes from cells grown under oxic conditions.
C. Analysis of co‐expression of *aliA‐badK*, *badH‐badI*, *aliB‐badH*, and *badI‐aabA* genes.

**FIGURE S3** Nucleotide sequence of the 440‐bp *aliA‐aliB* intergenic region. The ATG start codons of *aliA* (green) and *aliB* (red) genes are shown in boldface, and the predicted Shine‐Dalgarno sequences are underlined. The inferred −10 and − 35 boxes of the *P*_*aliA*_ and *P*_*aliB*_ promoters are indicated in green and red, respectively. The BadR operator regions in *P*_*aliA*_ and *P*_*aliB*_ are boxed in green and red, respectively.

**FIGURE S4** Growth of *E*. *coli* JW3375‐1 (Δ*bioH*) and *E*. *coli* JW3375‐1 containing plasmid pIZBad in MC minimal medium without biotin and using 0.2% glucose as carbon source. Cultures were amended with 3 mM CHC (as source of pimelyl‐CoA) and 1 mM IPTG (to induce the expression of the *bad‐ali* genes). Bacterial growth was monitored by measuring *A*_600_. Values are the mean of three different experiments. Error bars indicate standard deviations.

**FIGURE S5** Expression of *aab* genes and *AzCIB_2912* in *Aromatoleum* sp. CIB strains. Total RNA was isolated from *Aromatoleum* sp. CIB cells grown anaerobically in MC medium containing 3 mM benzoate (Bz), 3 mM CHC, or 3 mM pimelate (Pim) as sole carbon source until the end of the exponential phase. The expression of genes *aabD* (panel A), and *aabA* (panel B) was monitored by RT‐PCR using the oligonucleotide pairs detailed in Table S1. Agarose gel electrophoresis of RT‐PCR products is shown. Lane M, molecular size markers (Quick‐Load™ 100 bp DNA Ladder from New England BioLabs). Lanes Bz, CHC and Pim indicate RT‐PCR reactions using RNA isolated from cells grown with Bz, CHC, and Pim, respectively. Panel C shows the expression of gene *AzCIB_2912* (monitored by RT‐PCR using the oligonucleotide pair detailed in Table S1) in *Aromatoleum* sp. CIB (CIB) and *Aromatoleum* sp. CIBΔAzCIB_1938 (Δ1938) strains grown anaerobically in MC medium containing 3 mM CHC (CHC) or 3 mM Bz (Bz) as sole carbon source.

**TABLE S1** Oligonucleotides used in this work.

## Data Availability

Some of the data that support the findings of this study are available in the supplementary material of this article.
