# Supplementary material for: Genetic characterization of the cyclohexane carboxylate degradation pathway in the denitrifying bacterium Aromatoleum sp. CIB
Source: Environ Microbiol. 2022 Jun 29;24(11):4987–5004. doi: 10.1111/1462-2920.16093 (PMC9795900; doi:10.1111/1462-2920.16093)
Supplement: Supplementary file 1 — FIGURE S1 Nucleotide sequence of the synthetic bad‐ali cassette. The edited sequence of the aliB*(pink), aliA* (red), badK* (green), badH* (blue) and badI* (orange) genes is shown. The optimized Shine‐Dalgarno sequences with stop codons in all three reading frames and some additional restriction sites (EcoRI and SpeI) flanking the cassette are indicated in black. [file EMI-24-4987-s004.pdf]

*Eco*RI
AACCCCGAATTCTGACCTAAGGAGGTAAATAATGGATTTCAGTCTCAGTCCCGAGCAGCAAGCGCTCGTC
GACACCGCAAGCCGTTTCTCCCGCGAGCGTCTCGCTCCCGGCTACAAAGCACGCGAAAAGGCCGAACG
CATCGAGCGCGAAGTGATCCGCGAAATGGGCGAGCTGGGCTTCCTCGGACCGGAGCTGCCCGAGGAGC
ATGGCGGCATGGGGGTCGACTGCCTGACCAGCGGCCTGCTGCTGGAGCAGATCTCGTATGGCGACTTCA
ACGTCTCCTACGTGAATCTGCTCACCTCGCTGTGCGGCCAGATCGTCGCGAACTACGCGCAGCCGGACA
TCGCCAAGGAGTGGCTGGGCCAGGTCATCGCCGGCAAGAAGTCGATCGCCATCGCGCTGACCGAACCC
AGCGCCGGCTCCGACGCAGCGCGCCTGAAGCTGAAGGCGACGCGTGACGGTGACGCGTGGGTGCTCA
ACGGCGAGAAGACCTCGATCTCGATGGCGACGCAGGCGGATGTGGCGGTCGTGTTCGCCCGCACCGGC
AGCGAAGCCGATCGTGCTCGCGGCATCAGCGCCTTCCTGGTGCCGATGGATCTGCCGGGCATCACCCG
CACGGCTTTCGACGACATCGGCACCCGCCCGGTGGGCCGCGGCTCGATCTTCTTCGACGACGTGCGTG
TGCCGGCCGAAATGATGCTCGGCGACGAGGGCAAGGGTTTCGTTCAGGTGATGCAGGGCTTCGACTACA
GCCGCGCGCTCATCGGCATCCAGTGCATGGGCGTGGTGCGTGCCTCGCTCGACGAGACCTGGCGCTAC
GTGCAGGAACGCGAGGCCTTCGGCAAGAAGATCGGCGAGTTCCAGGGCGTGACTTTCCCGCTCGCCGA
AGCGGAGACGATGTACGAGGCATGCCGCGCGCTATGCCTGAAGACGCTGTGGCTGAAGGATCAGGGCC
TCGAGCACACGGCGGAAGCCGCGATGTGCAAATGGTGGGCGCCCAAGCTGGCGTGCGAAATCATTCAC
CAGTGCCTGCTGACGCATGGCCACGGCGGCTATGCGAGCGACTACGACTTCGGTCAGCGGTATCGCGAC
GTGATGGGTCTGCAGATCGGCGACGGCACCGCGAACATCATGAAGATGATCATCGGCCGCCAAAAGCTC
GCAGCGCACGAGATCTAATGACCTAAGGAGGTAAATAATGAATTTCGACCCGGTGCTGATCGCCGACCGC
ATGACTTCAATGAAGGCCGCGGGCCTGTGGCGCAACGAGACGATCGACGTGCATTTCCAGCGTGCGCTG
GAGAACTGCCCCGACAAGCTGGCCGTAGTGGCATACCGCGACGGCCAAGCCGAAGCGGCCCGTCTCAG
CTATCGCGAACTGGATCGACGCGTCGACCTGATCGCCCGCAATCTCGTCGCACTGGGCGTGGGCCGCA
GCGACGTCGTCAGCTTCCAGCTGCCCAACCGCTGGGAGTTCGTCGCGCTTTCCCTCGCCTGCGCCCGA
ATCGGTGCCGCTGCGAACCCCGTGATGCCGATCTTCCGTCAGCATGAACTCACCTACATGCTGAACTTCG
CCGAGACCAAGGTCTTCATCGTGCCGAGCGTGTTCCGCAAGTTCGACCACGCGGCGATGGCACGCGAA
CTACAGCCCAAACTGCCCCATCTGAAGCAGGTCGTCGTCGTGGACGGCGACGGGGAAGACAGCTTCGA
CCGCGTGCTGATGCGCGACGACACGCCGCCCCTTGCCGGCCTTGGCCTCGGACCCGACGACGTGTCG
CTCCTCATGTACACGTCTGGCACAACGGGCGAGCCCAAGGGCGTGATGCACACGTCCAACACGCTCTTC
TCGAACCTGCATGCGTACATCGCGACGATGGAGCTGGGCTCCTCGGACGTCGTCCTCGGCGCTTCGCCG
ATGGCTCACCTCACCGGCTACGGCTACCTCGCGATGCTGCCGCTGATCCTGAACTCGACGACCGTGCTG
CAGGAGATCTGGGACCCCGCGCGCGCACTCGAGATCGTCCGTGACGAGGGCGTGACCTTCAGCATGGC
CTCGACCGCCTTCATCTCCGACCTGTGCGCGGCGGTCGAAGCGGGCGCCCCGGTCTCGCCGCAATTCA
CCAAGTTCAACTGCGCCGGCGCACCCATCCCGCCGGTCGTCGTCCAGCGCGCCTGGGAGCTGATGGGG
CTGCGCGTGTGCTCGGCCTGGGGCATGACCGAATGCGGCGCCGTCACGATCACCGAACCCGTCCGTGC
GCTTGAGAAGTCTGGCGTCTCGGACGGCCGCGCCCTTCCGGGCATCGAGGTCCGCATCATTGACGCGA
ACGGCGACGAGGTACGCACCGGCGAGACGGGGGAACTGCTGATCCGCGGCTCGTCGCTGTTCGCCGG
GTATCTCAAGCGTCCCCAGCTAAACAGCGTGGACGCGAACGGCTGGTTCGACACCGGTGACCTCGCCTT
CCAGGACGGCGAGGGCTACATCCGCATCAACGGCCGCAGCAAGGACATCGTGATCCGCGGCGGCGAGA
ACATCCCGGTCATCGAGATCGAGAACCTGCTCTACCGGCACCCGTCCATCACCACGGTTGCGGTCGTCG
GCTACCCGGATCGCCGTCTGGGCGAGCGCGTGTGTGCCTTCGTCTCGCTGAAACCCGGCTGCACGCTG
ACTTTCGAGGACCTCACGGCCTATCTCGACAAGCAGCAGGTCGCCAAGCAGTATTACCCGGAGCGCCTC
GAGATCGTCGAAGACCTGCCCCGCACGCCGGCCGGCAAGCTGCAGAAATTCAAGCTGCGCGAGACCGC
CAAGTCCTTCGGCAACGACAAGTGACCTCAGCAGCTCGCCCCCATATGTGACCTAAGGAGGTAAATA
ATGGCGACGGGAGACGACACGATGATCTCGGTGATCCTCACTGAAACGCGCGGCAAGGTCGGCCTTATC
CGCATCAACCGCCCGGAAGTCTTCAACGCGCTCAACGATGACGTGATGACCGGGATCGGCCAGGCGCTC
GACCGCTTCGAAGCGGACCCCGCCATCGCTTGCGTCGTGGTGACGGGCTCCGAGAAGGCCTTCGCCGC
CGGTGCGGACATCGCCGCGATGCGCACGATGGACTACATGGACGCGTACAAGTCCGACTTCATCACGCG
CAACTGGGAGCGTCTCAAGACCTTCCGCAAGCCGACCATCGCCGCCGTCTCGGGCGTCGCGCTGGGCG
GCGGATGCGAACTCGCGATGATGTGCGACATCGTCTTCGCCGCTGAAAATGCCCGCTTCGGGCAGCCGG
AGATCAAGATCGGCACGATCCCCGGTGCAGGCGGAACGCAGCGCCTGCCGCGCGCCGTGGGCAAGAC
CAAGGCGATGGACCTGTGCCTGACCGGTCGCCTGATGGACGCGCAGGAAGCGGAGCGCAGCGGCCTG
GTCGCGCGCATCTACCCGCCCGAGAGCGTGCTCGACGAGGCGCTCGCAGCAGCCGCGAAGATCGCCGA
GTTCTCGCTGCCCGTCCTCATGATGATGAAGGAAACGGTGAATCGTGCCTTCGAAGGCCCGCTCAACGA
GGGCCTGCTGTTCGAGCGCCGCACGCTGCACGCGACCTTCGGGCTGGCGGACCAGAAGGAAGGGATG
AGCGCCTTCGTCGACAAGCGCCCGCCCCGCTTCGCCGATCGCTGATGACCTAAGGAGGTAAATAATGAG
AGGTCTTGAAGGCAAGGTCGTGATCGTGACGGGTGGTGCCGGTGGCATCGGTTCCGCAATCTGCCGCC
GCTTCGGCGAGGAGCGTGCGAGCGTCGCGGTGTTCGACATCAACCGCGAAGCGGCCGAAGCCGTTGTA
GCGGAAATCCAGTCCGCCGGCGGCAAGGCGCGTGCCTATGCGGTCGATCTGACGAGCCAGGATTCGGT
GATCTCGGCCGTAACGGCCGCCGAAGCTGAACTCGGCCCGATCGACGTGCTGGTGAATAACGCCGGCT
GGGACAAGGTCGGCAACTTCCTCGATACCGAGAAGCCGCTGTGGGACAAGATCGTGGCGATCAACCTGT
ACGGCGCTCTGTACATGCACCACGCGGTCGTCAAGGGCATGCGCGAGCGCGGCCGTGGCCGTGTCATC
AACGTCGCGTCCGACGCCGGCCGTGTCGGCTCGTCCGGCGAGGCGGTGTACTCGTTCTGCAAGGGCG
GCCTGATCTCGTTCTCGAAGACGCTGGCCCGCGAAGTCGCCCGTCAGCAGATCAACATCAACGTCGTGT
GCCCCGGCCCGACCGACACCCCGCTGCTGGATGACATCTGCGGCGAAGGCGAGCGCGGCGAGAAGCT
CCGTACCGCCTTCACCCGCGCCGTGCCCTTCGGCCGTCTCGGCCAGCCGGGCGACCTGGCTGGTGCC
GTGACCTTCCTCGCCAGCGACGACGCCGCGTTCATCACCGGTCAGGTGATCAGCGTGTCGGGCGGCCT
GACGATGGCCGGTTAAGCACCCAATTCACGACAACGACAAGGAGCAGAACATGGAATACCAGGACATTCT
CTACACCAAGCAGGACGGCATCGCGACCGTTACGATCAACCGCCCCGCCCAGTACAACGCCTTCCGCGC
CCAGACCTGCGAGGAGATGATCCACGCGCTCAAGGATGCGGATTACGACCGCAGCATCGGCGTGGTCGT
CCTGACCGGCGCCGGCGACAAGGCCTTCTGCACCGGCGGTGACCAGGGTACGCAGGACGGCGGCTAC
GGTGGCCGCGGCGTGATCGGCCTGCCGATCGAGGAAGTGCAGAGCGCCATCCGCGACATCTCCAAGCC
CGTGATCGCTCGCGTGAACGGCTTTGCGATCGGCGGCGGCAACGTGCTCGTGACGATCTGCGACCTCG
CCATCGCGTCCGACAAGGCCCAACTCGGCCAGGCCGGTCCGCGCGTCGGCTCGGTCGACCCCGGCTT
CGGCACCGCGCTGCTGGCGCGTGTGGTCGGCGAGAAGAAGGCGCGCGAGATCTGGTATCTGTGCCGCC
GCTACAACGCGCAGGAAGCGCTCGCGATGGGCCTCGTCAATGCCGTCGTGCCGCACGACCAGCTCGAC
GCCGAAGTGAAGAAGTGGTGCGACGAGATCGTCGAGAAGAGCCCGACCGCAATCGCGCTGGCGAAGAA
GTCGTTCAACGTCGATACGGAAATGATCCGCGGCATGGGCGGCCTGGCGATGCACGCGCTCAAGCTCTA
CTACGAGACGGCCGAGTCGGCCGAAGGCGGCAATGCCTTCCGCGAGAAGCGCAAGCCCGAGTTCCGCA
AGCACCAGAAGTAACCCGGGACTAGTATATGCC
*Spe*I
