# Supplementary figures and images for: Genetic characterization of the cyclohexane carboxylate degradation pathway in the denitrifying bacterium Aromatoleum sp. CIB
Source: Environ Microbiol. 2022 Jun 29;24(11):4987–5004. doi: 10.1111/1462-2920.16093 (PMC9795900; doi:10.1111/1462-2920.16093)

**A**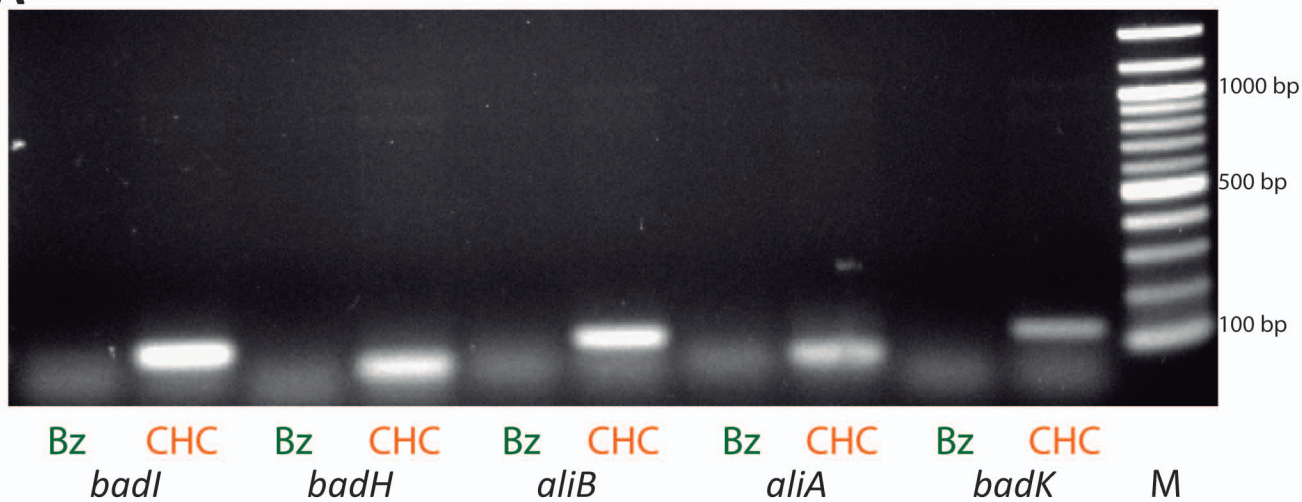**B**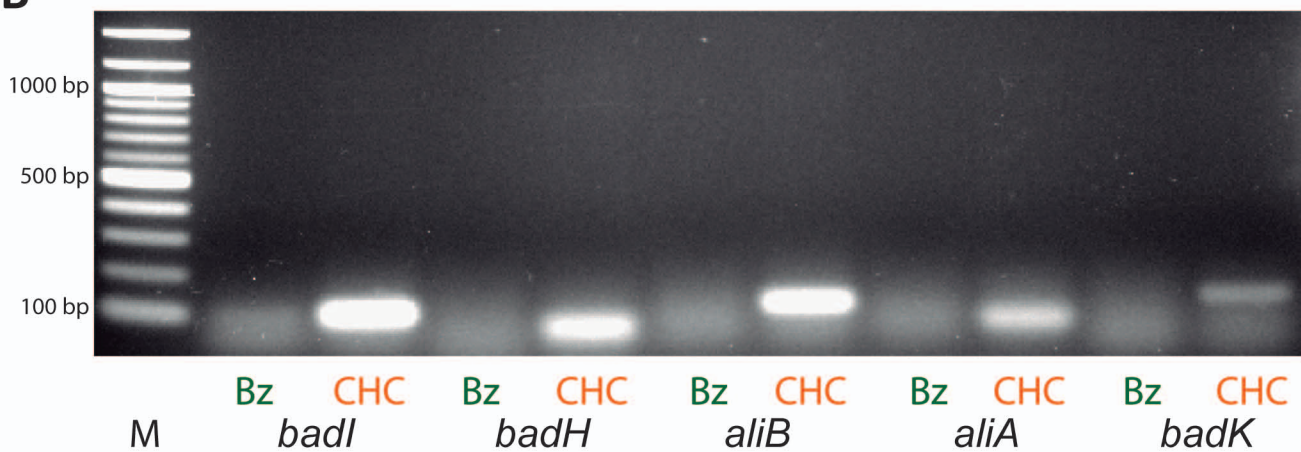**C**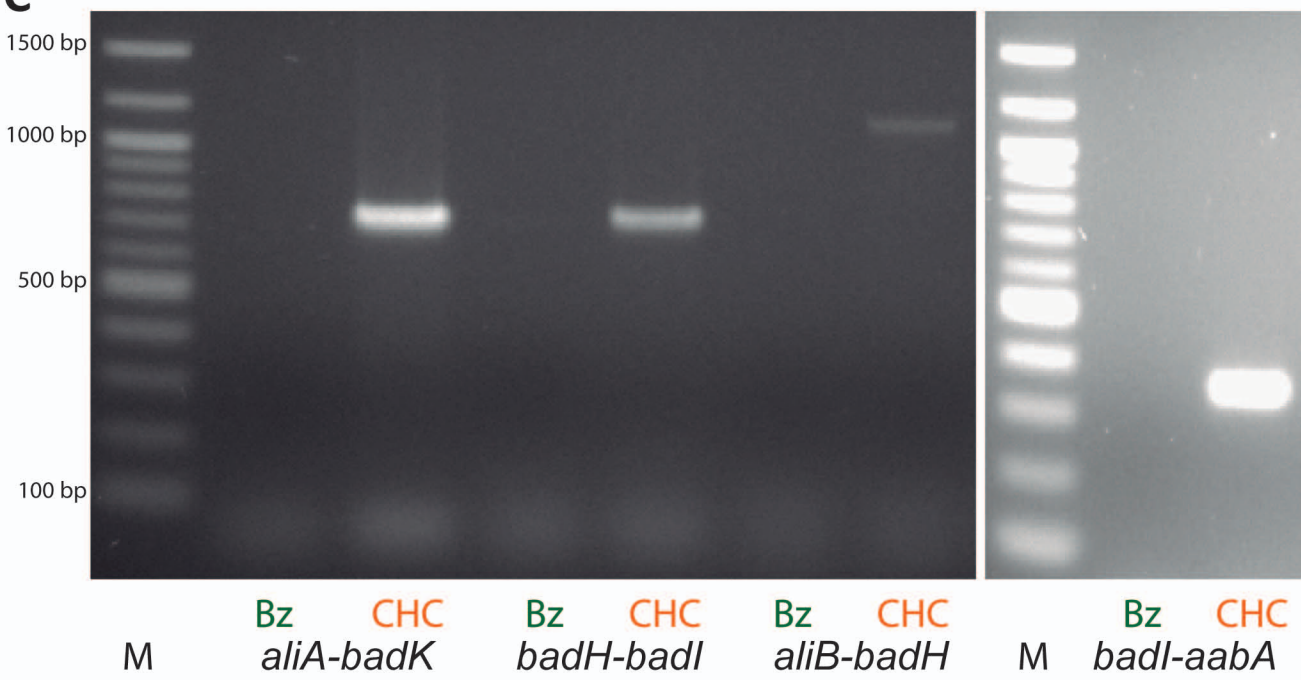

Supplement: Supplementary file 2 — FIGURE S2 Analysis of the expression of the bad‐ali genes in Aromatoleum sp. CIB. Total RNA was isolated from cells grown in MC medium containing 3 mM benzoate (Bz) or 3 mM CHC as sole carbon source until the end of the exponential phase. The expression of the target genes and intergenic regions was monitored by RT‐PCR using the oligonucleotide pairs detailed in Table S1. Agarose gel electrophoresis of RT‐PCR products is shown. Lane M, molecular size markers (Quick‐Load™ 100 bp DNA Ladder from New England BioLabs). Lanes Bz and CHC indicate RT‐PCR reactions from RNA isolated of cells grown with Bz and CHC, respectively. A. Expression of badI, badH, aliB, aliA and badK genes from cells grown under anoxic conditions. B. Expression of badI, badH, aliB, aliA and badK genes from cells grown under oxic conditions. C. Analysis of co‐expression of aliA‐badK, badH‐badI, aliB‐badH, and badI‐aabA genes. [file EMI-24-4987-s003.pdf]

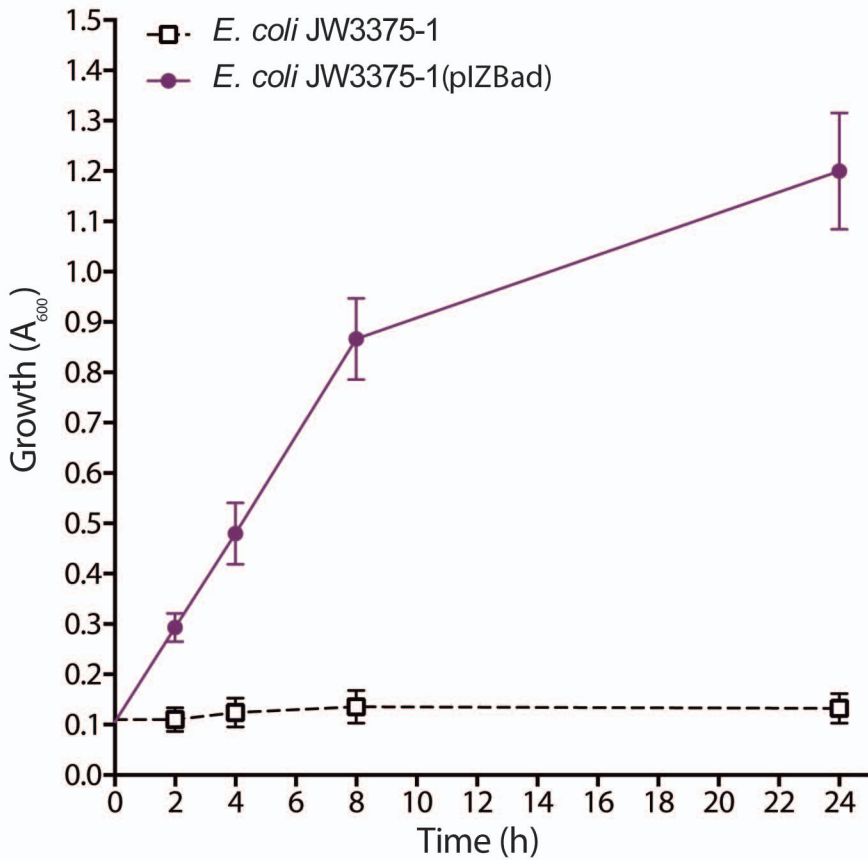

Supplement: Supplementary file 4 — FIGURE S4 Growth of E. coli JW3375‐1 (ΔbioH) and E. coli JW3375‐1 containing plasmid pIZBad in MC minimal medium without biotin and using 0.2% glucose as carbon source. Cultures were amended with 3 mM CHC (as source of pimelyl‐CoA) and 1 mM IPTG (to induce the expression of the bad‐ali genes). Bacterial growth was monitored by measuring A 600. Values are the mean of three different experiments. Error bars indicate standard deviations. [file EMI-24-4987-s005.pdf]

**A***aabD*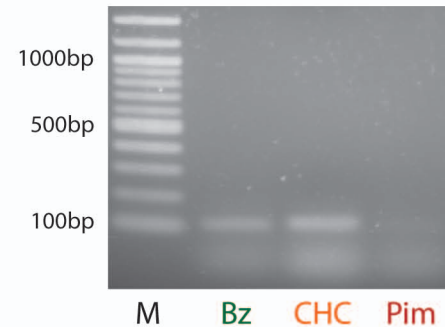**B***aabA*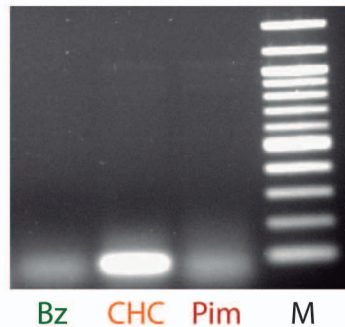**C***AzCIB\_2912*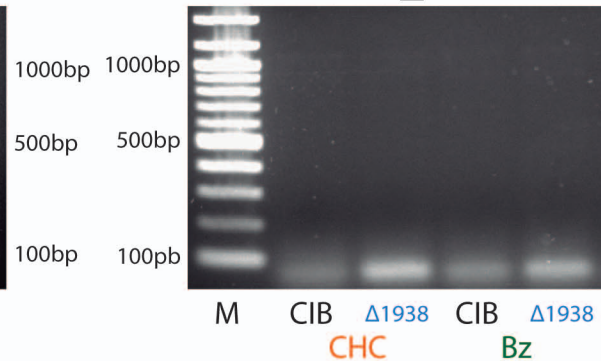

Supplement: Supplementary file 5 — FIGURE S5 Expression of aab genes and AzCIB_2912 in Aromatoleum sp. CIB strains. Total RNA was isolated from Aromatoleum sp. CIB cells grown anaerobically in MC medium containing 3 mM benzoate (Bz), 3 mM CHC, or 3 mM pimelate (Pim) as sole carbon source until the end of the exponential phase. The expression of genes aabD (panel A), and aabA (panel B) was monitored by RT‐PCR using the oligonucleotide pairs detailed in Table S1. Agarose gel electrophoresis of RT‐PCR products is shown. Lane M, molecular size markers (Quick‐Load™ 100 bp DNA Ladder from New England BioLabs). Lanes Bz, CHC and Pim indicate RT‐PCR reactions using RNA isolated from cells grown with Bz, CHC, and Pim, respectively. Panel C shows the expression of gene AzCIB_2912 (monitored by RT‐PCR using the oligonucleotide pair detailed in Table S1) in Aromatoleum sp. CIB (CIB) and Aromatoleum sp. CIBΔAzCIB_1938 (Δ1938) strains grown anaerobically in MC medium containing 3 mM CHC (CHC) or 3 mM Bz (Bz) as sole carbon source. [file EMI-24-4987-s002.pdf]
