# Supplementary material for: Genetic characterization of the cyclohexane carboxylate degradation pathway in the denitrifying bacterium Aromatoleum sp. CIB
Source: Environ Microbiol. 2022 Jun 29;24(11):4987–5004. doi: 10.1111/1462-2920.16093 (PMC9795900; doi:10.1111/1462-2920.16093)
Supplement: Supplementary file 3 — FIGURE S3 Nucleotide sequence of the 440‐bp aliA‐aliB intergenic region. The ATG start codons of aliA (green) and aliB (red) genes are shown in boldface, and the predicted Shine‐Dalgarno sequences are underlined. The inferred −10 and − 35 boxes of the P aliA and P aliB promoters are indicated in green and red, respectively. The BadR operator regions in P aliA and P aliB are boxed in green and red, respectively. [file EMI-24-4987-s006.pdf]

(*aliA*)**CAT**CGTTATCTCTCCTCTCGTGGCGGGGGCGTGGCCCGCACAACCCTGAATGTCGTCTTGC

GAGACGGCAGCGCTTCGGTCGCAGCGACGACGGCGACGCGTCGGCGTGGAAATGCGGTT

CGGCAATGCCTGTTGCGCCCGGTTTACCTGGGCACGTCTCGCTCGTTGATGTTGGCCCCCG

GCCTTCGCCGATGGCGACGCAAAGCTATTATCTTTGCAGAAGTAAGTCAATAGATTGTCGCAA

-10 -35

CGGCGGGTATGCGTGGCTGGCTGTGCTGGCTTGTGGTTCGCTGTAAAAATCTGTGCAGAAA

GGGCGGCGGGCCTTTTTGGTGTTGGTTGGTGATGAACCCAGTTTTGGCCGGTTAAAAAAAG

AAATAGCAATGTATTGACAGATAAGTTCGATGCTCCCTAGAATGCGCTCAACCTACACAGGAG

-35 -10

GAGCAGCG**ATG**(*aliB*)
