# Supplementary material for: Genetic characterization of the cyclohexane carboxylate degradation pathway in the denitrifying bacterium Aromatoleum sp. CIB
Source: Environ Microbiol. 2022 Jun 29;24(11):4987–5004. doi: 10.1111/1462-2920.16093 (PMC9795900; doi:10.1111/1462-2920.16093)
Supplement: Supplementary file 6 — TABLE S1 Oligonucleotides used in this work. [file EMI-24-4987-s001.pdf]

SUPPORTING INFORMATION

Genetic characterization of the cyclohexane carboxylate degradation pathway in the denitrifying bacterium *Aromatoleum* sp. CIB

David Sanz, and Eduardo Díaz *

Department of Microbial and Plant Biotechnology, Centro de Investigaciones Biológicas Margarita Salas-CSIC, Ramiro Maeztu 9, 28040 Madrid, Spain

***Corresponding author:**

Eduardo Díaz

Environmental Microbiology Group, Department of Microbial and Plant Biotechnology, Centro de Investigaciones Biológicas Margarita Salas-CSIC, Ramiro Maeztu 9, 28040 Madrid, Spain

Tel. (+34) 918373112

Fax: (+34) 915360432

Email: ediaz@cib.csic.es

Running title: The bad-aab *gene clusters in* Aromatoleum *sp. CIB*

Table S1. Oligonucleotides used in this work.

Primer	Sequence	Use
Δ*badHI* Fw 1 *Xba*I	CCAATCTAGAGCGAGCGACTACGACTTCGG (*Xba*I)	PCR-amplification and cloning of the flanking regions of the *badH* (719 bp) and *badI* (939 bp) genes to generate plasmid pK18mobsacBΔbadHI.
Δ*badHI* Rv 1 *Bam*HI	CCAAGGATCCCGCAAGCACCAGAAGTAAGCC (*Bam*HI)	
Δ*badHI* Fw 2 *Bam*HI	CCAAGGATCCGCCTTCAAGACCTCTCATTTTGC (*Bam*HI)	
Δ*badHI* Rv 2 *Hin*dIII	GGCCAAGCTTCTTGTCGAGCACCTTCATCGCGG (*Hin*dIII)	
Δ*AzCIB_1938* Fw 1 *Xba*I	AGGGGTCTAGACAGCGAAGCGGTCCGAATCC (*Xba*I)	PCR-amplification and cloning of the flanking regions of the *AzCIB_1938* gene (612 bp and 809 bp) to generate plasmid pK18*mobsacBΔAzCIB_1938*.
Δ*AzCIB_1938* Rv 1 *Nde*I	CCCCCCATATGCTGACGCCCGACAACTGAC (*Nde*I)	
Δ*AzCIB_1938* Fw 2 *Nde*I	GGGGGCATATGACGCATTGCATCGAACATCTC (*Nde*I)	
Δ*AzCIB_1938* Rv 2 *Hin*dIII	GGGGGAAGCTTCGAGATGGATGCAGTAGTCGTCG (*Hin*dIII)	
badR Fw *Xba*I	CCCCCTCTAGATGACCTAAGGAGGTAAATAATGAAAGACCTTTCCGCTGCC (*Xba*I)	Cloning of the *badR* gene (509 bp) from *Aromatoleum* sp. CIB in plasmid pIZ2 double digested with *Xba*I/*Hin*dIII, to generate plasmid pIZBadR. The amplified sequence includes an
badR Rv *Avr*II *Hin*dIII	CCCCCAAGCTTCCTAGGCTAAAGCCTGGCCATGTTTCGC	

	(*Hin*dIII)	optimized Shine-Dalgarno sequence with translation stop codons in all three reading frames (double underlined).
badR Fw pET *Nde*I	GGGGGGCATATGAAAGACCTTTCCGCTGCC (*Nde*I)	Cloning of the badR gene (477 bp) from *Aromatoleum* sp. CIB in plasmid pET-28a double digested with *Nde*I/*Hin*dIII, to generate plasmid pET-28BadR.
badR Rv pET *Hin*dIII	GGGGGGAAGCTTAAGCCTGGCCATGTTTCGC (*Hin*dIII)	
aliA Fw *Hin*dIII	CCCCCCAAGCTTTGACCTAAGGAGGTAAATAATGAATTTCGACCCGGTGCTG (*Hin*dIII)	Cloning of the *aliA* gene (1,672 bp) from *Aromatoleum* sp. CIB in plasmid pIZBadR double digested with *Hin*dIII/*Sac*I, to generate plasmid pIZBadRAliA. The amplified *aliA* gene includes an optimized Shine-Dalgarno sequence with translation stop codons in all three reading frames (double underlined).
aliA Rv *Sac*I	AAAAAAGAGCTCTCACTTGTCGTTGCCGAAGGAC (*Sac*I)	
P*aliB* Fw *Hin*dIII	CCCCCAAGCTTCTTGCGGTGTCGACGAGCGCTTGC (*Hin*dIII)	Cloning of the *PaliB* promoter (262 bp) from *Aromatoleum* sp. CIB in plasmid pSEVA225T double digested with *Hin*dIII/*Bam*HI, to generate plasmid pSEVA225TPaliB.
P*aliB* Rv *Bam*HI	AAAAAGGATCCTCGCAACGGCGGGTATGCGTGGC (*Bam*HI)	
AzCIB_1942 Fw *Spe*I	CCCCCACTAGTTGACCTAAGGAGGTAAATAATGATCCGCGACCAGGAGAC (*Spe*I)	Cloning of the *aabA* (*AzCIB_1942*) gene (1,198 bp) from *Aromatoleum* sp. CIB in plasmid pIZBad double-digested with *Spe*I/*Sbf*I to generate plasmid
AzCIB_1942	AAAAACCTGCAGGTCATTCGC	

Rv *Sbf*I	CTGCAGCCCGG (*Sbf*I)	pIZBad_A. The amplified *aabA* gene includes an optimized Shine-Dalgarno sequence with translation stop codons in all three reading frames (double underlined).
AzCIB_1939 Fw *Sbf*I	GGGGGCCTGCAGGTGACCTAAGGAGGTAAATAATGGATGCAGTAGTCGTCGAACG (*Sbf*I)	Cloning of the genes *AzCIB_1939*-*AzCIB_1937* (3,580 bp) from *Aromatoleum* sp. CIB in plasmid pIZBad_ A double -digested with *Sbf*I/*Hin*dIII to generate plasmid pIZBadβ1. The amplified *aabBCD* genes include an optimized Shine-Dalgarno sequence upstream of *aabB* (double underlined) and two new restriction sites, *Xba*I and *Mfe*I, downstream of *aabD*.
AzCIB_1937 Rv *Hin*dIII	CCCCCCAAGCTTCAATTGTCTAGATTCAGCCCGGAATGCGCTCG (*Hin*dIII)	
Bad Sec 1	TCGTTCAGGTGATGCAGGGC	Sequencing of the synthetic *bad* cluster in plasmid pIZBad.
Bad Sec 2	ATCGACGTGCATTTCCAGCG	
Bad Sec 3	TTGAGAAGTCTGGCGTCTCGG	
Bad Sec 4	CTTCGGCAACGACAAGTGACC	
BadHI- Fw	AAGAAGATCGGCGAATTCCAGGGCG	Checking deletion of *badH* and *bad*I genes in *Aromatoleum* sp. CIBΔbadHI
BadHI- Rv	GCAGCACATTTCCGAAGCGAAGAGC	
AzCIB_1938-	TCCGATCACGGCGTTGTCCAT	Checking deletion of *AzCIB_1938* gene

Fw	CACC	in *Aromatoleum* sp. CIBΔ1938
AzCIB_1938^{-} Rv	TCTTCCATTTCCCCACCTCCACGCG	
P*aliB* Fw EMSA WT *Eco*RI	GGGGGGGAATTCCGCTGCTCCTCCTGTGTAGGTTGAGCGCATTCTAGGGAGCATCGAACTTATCTGT (*Eco*RI)	5' end primer used to amplify a 100-bp fragment that includes the PaliB promoter and that was used for electrophoretic mobility shift assays (EMSA).
P*aliB* Rv EMSA WT	TGAACCCAGTTTTGGCCGGTTAAAAAAAGAAATAG**CAATGTATTG**ACAGATAAGTTCGATGCTCCC	3' end primers used to amplify a 100-bp PaliB promoter fragment harboring the parental BadR-binding site (P*aliB* Rv EMSA WT) or different mutant operators (bold and underlined), used for electrophoretic mobility shift assays (EMSA).
P*aliB* Rv EMSA CAATATTG	TGAACCCAGTTTTGGCCGGTTAAAAAAAGAAATAG**CAATATTG**ACAGATAAGTTCGATGCTCCC	
P*aliB* Rv EMSA CAATGTATTC	TGAACCCAGTTTTGGCCGGTTAAAAAAAGAAATAG**CAATGTATTC**ACAGATAAGTTCGATGCTCCC	
P*aliB* Rv EMSA CAACCCCTTG	TGAACCCAGTTTTGGCCGGTTAAAAAAAGAAATAG**CAACCCCTTG**ACAGATAAGTTCGATGCTCCC	
P*aliB* Rv EMSA CATTATATTG	TGAACCCAGTTTTGGCCGGTTAAAAAAAGAAATAG**CATTATATTG**ACAGATAAGTTCGATGCTCCC	
P*aliB* Rv EMSA CAATGTGT	TGAACCCAGTTTTGGCCGGTTAAAAAAAGAAATAG**CAATGTGTATTG**ACAGATAAGTTCGA	

ATTG	TGCTCCC	
P*aliB* Rv EMSA CTTTGTATTG	TGAACCCAGTTTTGGCCGGTTAAAAAAAGAAATAG**CTTTGTATTG**ACAGATAAGTTCGATGCTCCC	
P*aliB* Rv EMSA CAATGTGTGTATTG	TGAACCCAGTTTTGGCCGGTTAAAAAAAGAAATAG**CAATGTGTGTATTG**ACAGATAAGTTCGATGCTCCC	
F24	CGCCAGGGTTTTCCCAGTCACGAC	Sequencing of the DNA fragments cloned in pIZ1016 and pIZ2 plasmids.
R24	AGCGGATAACAATTTCACACAGGA	
T7	TAATACGACTCACTATAGGG	Sequencing of DNA fragments cloned into pET plasmids.
TT7	GCTAGTTATTGCTCAGCGG	
16S1	AAGGAGGTGATCCAGCC	16S rDNA sequencing to confirm the identity of the bacterial strains.
16S2	GAGASTTTGATCHTGGCTCAG	
1387 Rv	GGGCGGWGTGTACAAGGC	
63 Fw	CAGGCCTAACACATGCAAGTC	
aliA RT Fw	TTCCGCAAGTTCGACCAC	Amplification of an internal fragment (77 bp) of the *aliA* gene of *Aromatoleum* sp. CIB, used in RT-PCR assays.
aliA RT Rv	ACGACGACGACCTGCTTC	
badK RT Fw	ATGGACCTGTGCCTGACC	Amplification of an internal fragment (135 bp) of the *badK* gene of *Aromatoleum* sp. CIB, used in RT-PCR assays.
badK RT Rv	CGAGAACTCGGCGATCTT	
aliB RT Fw	AAGTGATCCGCGAAATGG	Amplification of an internal fragment (120 bp) of the *aliB* gene of *Aromatoleum* sp. CIB, used in RT-PCR
aliB RT Rv	CGCCATACGAGATCTGCTC	

		assays.
badH RT Fw	AGCCGTTGTAGCGGAAATC	Amplification of an internal fragment (64 bp) of the *badH* gene of *Aromatoleum* sp. CIB, used in RT-PCR assays.
badH RT Rv	CGTCAGATCGACCGCATAG	
badI RT Fw	GCGACCGTTACGATCAACCG	Amplification of an internal fragment (104 bp) of the *badI* gene of *Aromatoleum* sp. CIB, used in RT-PCR assays.
badI RT Rv	ATGCTGCGGTCGTAATCCG	
AzCIB_1942 RT Fw	TGTTCGGGCTGTCGATTC	Amplification of an internal fragment (88 bp) of the *aabA* gene of *Aromatoleum* sp. CIB, used in RT-PCR assays.
AzCIB_1942 RT Rv	GTGCGACCGATCTCGAAG	
AzCIB_1937 RT Fw	TCTACGCAGGGGAGATCCT	Amplification of an internal fragment (112 bp) of the *aabD* gene of *Aromatoleum* sp. CIB, used in RT-PCR assays.
AzCIB_1937 RT Rv	GCCAGCGACTCGTAGGTC	
aliA-badK CRT Fw	GCACGCTGACTTTCGAGGACC	Amplification of a 710 bp fragment between the *aliA* and *badK* genes of *Aromatoleum* sp. CIB, used in RT-PCR assays for co-transcriptional analysis.
aliA-badK CRT Rv	TGGCCGATCCCGGTCATCAC	
aliB-badH CRT Fw	TGATGGGTCTGCAGATCGGCG	Amplification of a 1135 bp fragment between the *aliB* and *badH* genes of *Aromatoleum* sp. CIB, used in RT-PCR assays for co-transcriptional analysis.
aliB-badH CRT Rv	GCAGATTGCGGAACCGATGCC	

badH-badI CRT Fw	GAAGCTCCGTACCGCCTTC	Amplification of a 721 bp fragment between the *badH* and *badI* genes of *Aromatoleum* sp. CIB, used in RT-PCR assays for co-transcriptional analysis.
badI RT Rv	ATGCTGCGGTCGTAATCCG	
badI-AzCIB_1942 CRT Fw	CTCTACTACGAGACGGCCGAG	Amplification of a 325 bp fragment between the *badI* and *aabA* genes of *Aromatoleum* sp. CIB, used in RT-PCR assays for co-transcriptional analysis.
badI-AzCIB_1942 CRT Rv	GCACGAAGCGCGAAATCGTG	
AzCIB_2912 RT Fw	GTGCATCCCCGAGGAATA	Amplification of an internal fragment (70 bp) of the *AzCIB_2912* gene of *Aromatoleum* sp. CIB, used in RT-PCR assays.
AzCIB_2912 RT Rv	GATCTCGAACGCGACCAG	
